# Inferring gene function from evolutionary change in signatures of translation efficiency

**DOI:** 10.1186/gb-2014-15-3-r44

**Published:** 2014-03-03

**Authors:** Anita Krisko, Tea Copic, Toni Gabaldón, Ben Lehner, Fran Supek

**Affiliations:** 1 Mediterranean Institute for Life Sciences (MedILS), 21000 Split, Croatia; 2 Bioinformatics and Genomics Programme, Centre for Genomic Regulation (CRG), Dr. Aiguader 88, 08003 Barcelona, Spain; 3 Universitat Pompeu Fabra (UPF), 08003 Barcelona, Spain; 4 EMBL/CRG Systems Biology Research Unit, Centre for Genomic Regulation (CRG), Dr. Aiguader 88, 08003 Barcelona, Spain; 5 Institució Catalana de Recerca i Estudis Avançats (ICREA), Pg. Lluis Companys 23, 08010 Barcelona, Spain

## Abstract

**Background:**

The genetic code is redundant, meaning that most amino acids can be encoded by more than one codon. Highly expressed genes tend to use optimal codons to increase the accuracy and speed of translation. Thus, codon usage biases provide a signature of the relative expression levels of genes, which can, uniquely, be quantified across the domains of life.

**Results:**

Here we describe a general statistical framework to exploit this phenomenon and to systematically associate genes with environments and phenotypic traits through changes in codon adaptation. By inferring evolutionary signatures of translation efficiency in 911 bacterial and archaeal genomes while controlling for confounding effects of phylogeny and inter-correlated phenotypes, we linked 187 gene families to 24 diverse phenotypic traits. A series of experiments in *Escherichia coli* revealed that 13 of 15, 19 of 23, and 3 of 6 gene families with changes in codon adaptation in aerotolerant, thermophilic, or halophilic microbes. Respectively, confer specific resistance to, respectively, hydrogen peroxide, heat, and high salinity. Further, we demonstrate experimentally that changes in codon optimality alone are sufficient to enhance stress resistance. Finally, we present evidence that multiple genes with altered codon optimality in aerobes confer oxidative stress resistance by controlling the levels of iron and NAD(P)H.

**Conclusions:**

Taken together, these results provide experimental evidence for a widespread connection between changes in translation efficiency and phenotypic adaptation. As the number of sequenced genomes increases, this novel genomic context method for linking genes to phenotypes based on sequence alone will become increasingly useful**.**

## Background

The genetic code is redundant, meaning that most amino acids can be encoded by more than one codon. Across diverse species, highly expressed genes tend to use optimal codons to increase the accuracy and speed of translation by ensuring better agreement with the cellular tRNA pools [1-3]. Consequently, codon biases are predictive of expression levels in both natural [4,5] and designed [6,7] gene sequences. This ‘translational selection’ acting to increase the use of optimal codons is stronger in faster growing microbes with large effective population sizes [8], but it has been shown to be widespread in both prokaryotes and eukaryotes [9-11], allowing the signatures of high gene expression to be detected and compared across species [12,13].

Interestingly, several previous studies have suggested a link between increased translation efficiency in specific groups of orthologous genes and phenotypic change during evolution [14-16]. Examples include increased codon optimization of photosynthesis genes in *Synechocystis* and methanogenesis genes in *Methanosarcina acetivorans*[14], reflecting their trophic preferences, and an increased use of optimal codons in glycolytic enzymes in anaerobic microbes or in the Krebs cycle in aerobes [15]. In nine yeast species, the same trend was observed [16] and, in addition, species adapted either to aerobic or anaerobic growth had consistently higher codon adaptation in the mitochondrial or cytoplasmic ribosomal protein (RP) genes, respectively. This correlation could not be explained by the phylogenetic distribution of (an)aerobes, indicating that mere genetic drift is not sufficient to drive the evolution of translation efficiency [16].

These examples of the coupling of codon usage to adaptive phenotypic variation suggest that it might be possible to systematically infer gene function from evolutionary change in the use of optimal codons. The basis for this argument is that diverse species sharing a common phenotypic trait, such as resistance to high temperature, might show increased expression, via a convergent codon adaptation, in a common set of genes involved in that phenotypic trait. However, four important challenges have so far prevented the large-scale inference of novel translation efficiency-phenotype links: 1) insufficient coverage with genomic sequences necessary to detect a weak evolutionary signal; 2) methodological issues with common approaches for predicting expression from codon biases in certain genomes [5,17,18], and with rescaling the predictions to make them comparable across genomes; 3) difficulties in disentangling the influences of the phylogeny and a particular phenotype; and 4) extensive correlations between different phenotypes. For instance, Archaea are typically obligate anaerobes, and within Bacteria, thermophiles tend to be less commonly aerotolerant than mesophiles. Thus, an observed correlation between a genomic feature and aerotolerance might be an artifact of either thermophilicity or phylogenetic relatedness.

Here, we explicitly address these issues using a novel statistical framework to identify meaningful correlations between phenotypes and signatures of selection for translation efficiency. Our approach generalizes over the previous explanatory models for a few select phenotypes to a broadly applicable framework that generates many testable predictions about the genes involved in adaptation to various environments. We experimentally validate a set of predicted gene-phenotype links for genes involved in three environmental adaptations: resistance to oxidative stress, heat, and high salinity. Moreover, we confirm experimentally that changing the codon usage of a gene can be sufficient to confer the expected stress resistance phenotype. Our approach therefore provides a potentially general strategy for annotating gene function in newly sequenced genomes by identifying genes whose translation efficiency is linked to particular phenotypes, important stress responses, or environmental adaptations.

## Results

### A novel method links translation efficiency of gene families to phenotypes

**Figure 1:**
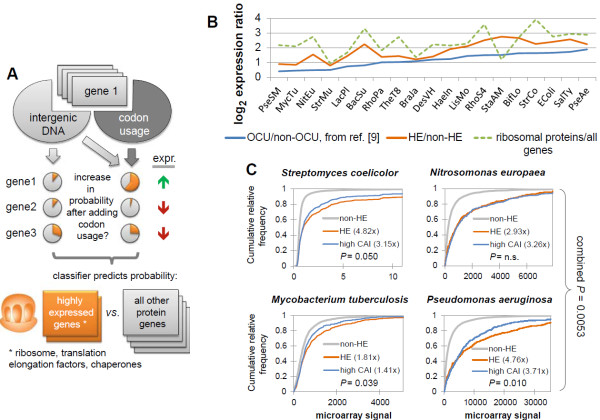
**Finding highly expressed (HE) genes in prokaryotic genomes and their enrichment in particular phenotypic groups. (A)** HE labels for genes were predicted by comparing the codon usage of each gene with that of a small set of known HE genes, while controlling for local background nucleotide composition determined from the neighboring intergenic DNA. **(B)** Comparison of average microarray signal intensity between the HE genes from this study, and the HE gene group of Supek *et al*. [9] in 19 diverse bacterial genomes, denoted by the UniProt species code on the *x* axis. The machine learning pipeline used here was derived from the methodology of Supek *et al*. [9] (see Additional file 1). **(C)** The predictions of HE genes compared favorably with those of the codon adaptation index (CAI) when evaluated against microarray data in four genomes previously claimed to lack detectable selected codon biases [21-23], representing difficult cases for predicting gene expression from codon usage. The *x* axes range from the minimum to the 99th percentile of microarray signal intensities. The farther away a curve is from the non-HE curve, the better the separation. Numbers in the parenthesis are the ratios of average expression in the HE (or high CAI) genes, compared with the non-HE genes. The ‘high CAI’ category was defined as containing the same number of genes as the HE category in each genome. *P* values are from a one-tailed Kolmogorov-Smirnov test for HE > high CAI, and were combined using Fisher’s method.

The codon usage of individual genes is to a large extent determined by mutational processes unrelated to translational selection [19,20], thus necessitating that these influences be factored out before predicting expression levels from codon biases. To this end, we used a machine learning-based method, which tests whether a given gene’s codon usage pattern is more similar to a reference set of highly expressed genes than would be expected from the background nucleotide composition in intergenic DNA [9]. Using this approach, we assigned a categorical high/low expression label to genes in 911 bacterial and archaeal genomes (Figure 1A). Changes in the methodology (see Additional file 1) substantially improved the agreement of the predictions with microarray data in 19 diverse bacterial species (Figure 1B; see Additional file 2). The predicted highly expressed genes (at a false discovery rate (FDR) of ≤10^-12^, sign test) comprise 4 to 20% of the genome, depending on the genome size (see Additional file 3), and have on average 3.9 times higher microarray signal levels than the rest of the genes (*P* = 10^-47^ by Mann–Whitney test, median of 19 genomes) (Figure 1B; see Additional file 2). For comparison, the very highly expressed ribosomal protein genes are 6.1 times above the genome average in the 19-genome dataset.

Genomes under weak selection for translation efficiency represent a difficult case for detecting signatures of expression levels in codon biases. In three out of four such genomes, the predictions from our machine learning-based method showed better correlation with mRNA levels than did those obtained by a commonly used approach, the CAI [4] (Figure 1C; combined *P* = 0.0053, one-tailed Kolmogorov-Smirnov test). Importantly, although gene expression levels may change substantially across different conditions, the genome-encoded codon biases are static, and are likely to reflect the gene expression in a typical environment encountered by the organism during evolution [24].

In addition to codon usage, other coding sequence determinants can shape protein levels. For instance, strong secondary structures at the mRNA 5′ end were shown to influence translation efficiency in a library of synthetic gene variants [25]. However, we found no correlation between mRNA 5′ folding free energies and gene expression levels in the 19 evaluated bacterial genomes (median *r* = 0.02 to 0.05; see Additional file 4), in contrast to various codon indices (median *r* = 0.22 to 0.43). This is consistent with mRNA folding being more relevant for highly stable 5′ mRNA structures [7], which we found to occur only infrequently in real genomes (median 13 to 17% of genes, depending on their position in the mRNA; see Additional file 4).

**Figure 2:**
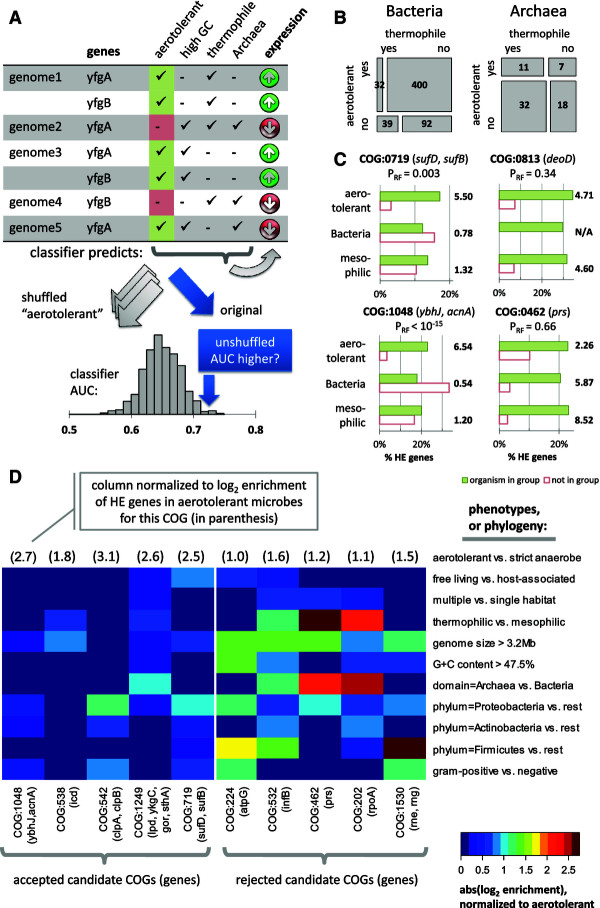
**Enrichment for highly expressed (HE) genes in gene families across microbial phenotypic groups. (A)** Phenotypes were tested for an independent contribution to predicting expression levels within a gene family, after controlling for 24 other phenotypes, 6 genomic descriptors, and 70 phylogenetic subdivisions using a Random Forest (RF) randomization test (see Additional file 1). **(B)** An example of correlation between two phenotypes (here, thermophilicity and aerotolerance), and their correlation with taxonomy. The area of the rectangles is proportional to the number of genomes in each subgroup (overlaid). **(C)** Enrichment with HE genes in four example clusters of orthologous groups (COG) gene families in aerotolerant microbes versus obligate anaerobes, compared with HE enrichments in two other aerotolerance-correlated traits: genomic G + C content and thermophilicity. The ‘accepted’ COGs (left) have stronger HE enrichments for aerotolerance than for the other traits, whereas the HE enrichment in the ‘rejected’ COGs (right) can be more easily explained both by the aerotolerance and by another trait. **(D)** Enrichment of example COGs with HE genes in 10 groups of microbes defined through phenotypic traits, genomic features (GC, size) or taxonomy. The COGs shown all have *Escherichia coli* representative genes, and were found to have at least a twofold enrichment in HE genes in aerotolerant microbes compared with obligate anaerobes (*P* < 0.01, Fisher’s exact test). Left block shows the five HE-enriched genes with the most significant *P*-values in the RF randomization test for confounding phenotypes/phylogeny, while the right block shows the genes with the least significant *P*-values in this test. The more significant COGs tended to be less HE-enriched in other phenotypes or phylogenetic groups relative to the HE enrichment in aerotolerant microbes. Thus, the aerotolerant phenotype contains the information about the HE enrichment of genes within these particular COGs that cannot be recovered from the other traits.

To infer whether increased or reduced translational efficiency of a gene is adaptive in a particular environment or is associated with a particular phenotype, we searched for correlations between the high/low expression levels of orthologous gene groups (as identified in clusters of orthologous groups (COGs) [26]) and the phenotypes or environments annotated to each species. We used a statistical framework based on supervised machine learning that searches within a large set of translation efficiency-phenotype correlations to find the phenotypes that contribute independently to the prediction of translation efficiency, after controlling for all the confounding phenotypes or taxonomic subdivisions (see Additional file 5; summarized in Figure 2A; examples of confounders in Figure 2B). Our method provides predictions for 187 gene families (COGs), which are linked to 24 different phenotypes (see Additional file 5), including the ability to colonize various environments, and plant and mammalian pathogenicity (200 predictions in total).

In 71 of 911 genomes, the detected codon bias did not fully match the optimal codons predicted from genomic tRNA gene composition (see Additional file 6), and it is thus not clear whether translational selection causes the observed signature of high expression in these genomes. tRNA modifications have been hypothesized as a cause for such discrepancies [9,27]. Upon excluding the 71 genome set, we found that our 200 phenotype predictions were highly robust to this factor (see Additional file 7).

Next, for three selected phenotypes, we evaluated these predictions by performing experiments in a series of *Escherichia coli* deletion mutants.

### Genes with altered codon adaptation in aerobes protect *E. coli* against oxidative stress

We first focused on genes with differential translation efficiency signatures between 514 aerotolerant microbes and 214 obligate anaerobes. We found that 295 COGs had a significant change in the content of highly expressed (HE) genes (at least twofold enrichment, FDR = 9.6% by Fisher’s exact test). Of these, only 23 COGs passed a control to ensure that the enrichment for HE genes could not be explained by the 23 other phenotypes, the 6 genomic features, or the 70 taxonomic subdivisions (*P* < 10^-2^, Random Forest permutation test). The percentages of HE genes for four example COGs passing or failing this test are shown for select phenotypes in Figure 2C, and the enrichments for a broader set of COGs and phenotypes are shown in Figure 2D. Similarly, a comparison between 296 obligate aerobes and 217 facultative aerobes identified 160 differentially expressed COGs (FDR = 11.8%), with 11 COGs remaining after controlling for confounding factors. In total, 34 differentially expressed COGs were found for the two oxygen-related phenotypes.

**Figure 3:**
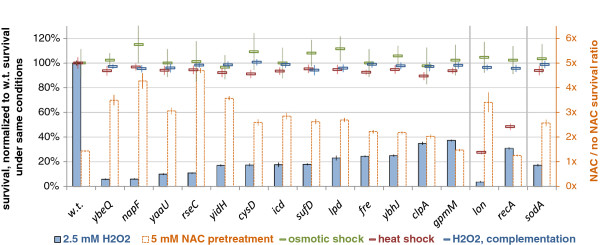
**Survival of *Escherichia coli *deletion mutants in genes where codon adaptation was linked to aerobicity.** H_2_O_2_ shock survival of mutants in putative oxidative stress genes (those with changes in codon adaptation in aerotolerant or obligately aerobic microbes, false discovery rate (FDR) ≤9.6% and ≤11.8%, respectively), without or with pre-treatment with N-acetylcysteine (NAC). More H_2_O_2_ concentrations are shown in Additional file 8. Deleted genes are *E. coli* representatives of clusters of orthologous groups (COGs) with codon adaptation correlated with oxygen in the environment, after controlling for confounding phenotypes or taxonomy. Strains are ordered by H_2_O_2_ survival, normalized to the wild-type survival under the same stress (13.8% for H_2_O_2_ after normalization shown as 100% on the plot). The outcome of the NAC rescue experiment is shown as a fold change in H_2_O_2_ survival over the same strain without NAC (right *x* axis). Additionally, the survival of each strain after heat and osmotic shocks is given for comparison; normalization as above. The strains *lon* and *recA* showed non-specific sensitivity and were thus separated on the plot, alongside *sodA*, which was included as a positive control. Error bars show the 95% C.I. of the mean, determined over at least four replicates.

Of the 34 COGs, 22 were present in the *E. coli* MG1655 genome, and 15 of these had viable deletion mutants. These *E. coli* strains comprised a biological model system for testing the hypothesis that genes with differential codon adaptation in microbes exposed to varying oxygen levels have a role in resisting the oxidative stress associated with the aerobic lifestyle. All 15 *E. coli* deletion mutants exhibited higher sensitivity to hydrogen peroxide exposure than the wild-type strain (Figure 3A). In particular, nine mutant strains were similarly or more sensitive to 2.5 mM H_2_O_2_ than the *sodA* strain lacking the Mn-containing superoxide dismutase (≤20% of wild-type survival). Decreased survival of the 15 mutants was observed across a range of H_2_O_2_ concentrations that spanned almost two orders of magnitude (0.5 mM to 20 mM; see Additional file 8).

To verify that the deletions caused sensitivity to oxidative stress specifically instead of a general frailty of the bacteria, we exposed the mutants to heat and osmotic shock, and found that 13 strains were as resistant as the wild type (≥90% of wild-type survival, Figure 3A). The two remaining non-specifically sensitive strains were *recA*, deficient in the SOS response and in recombination DNA repair, and *lon*, lacking a major protease dealing with clearance of oxidized proteins. Both mutants are known to be sensitive to a variety of different stresses.

**Figure 4:**
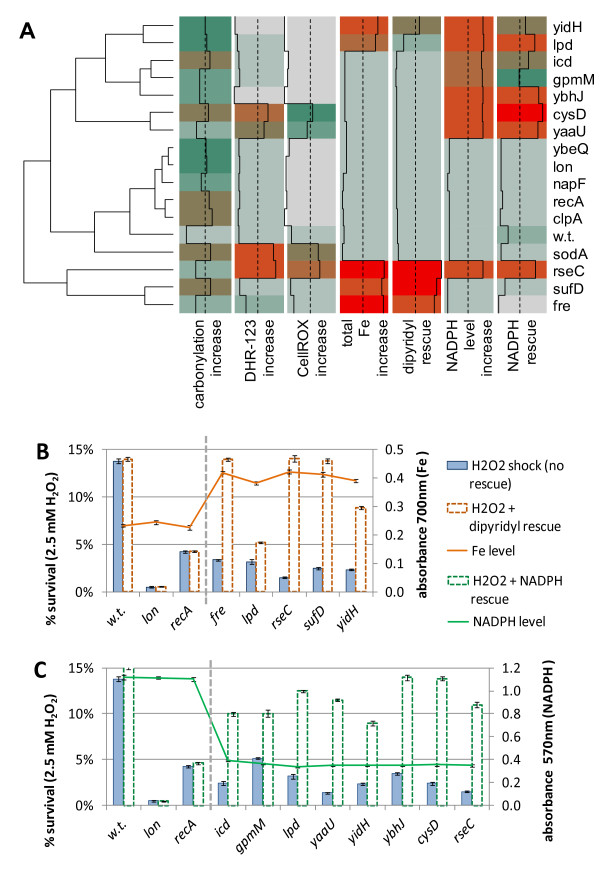
**Mechanism of activity of the putative oxidative stress protection genes. (A)** Overview of outcomes of seven experimental assays (columns) performed with the wild-type *Escherichia coli* and 15 deletion mutants. A larger value denotes a stronger observed effect; values are adjusted so that 0 signifies no effect and 1 signifies strong effect (values <0 and >1 are possible; for details of normalization for each assay, see Additional file 1). DHR-123 and CellROX are fluorescent dyes that measure reactive oxygen species. 2,2′-dipyridyl is an iron chelator. Genes are clustered based on similarity of the normalized response profiles of the mutants across the assays. Dashed lines denote the median. **(B, C)** A detailed display of the non-normalized measurements of: **(B)** iron levels and survival in the dipyridyl rescue experiment, or **(C)** NADPH levels and survival in the corresponding rescue experiment. Data are shown for wild-type *E. coli*, for *lon* and *recA* mutants (well-investigated genes expected not to act by the examined mechanisms), and those candidate genes in which our experiments support the proposed mechanism of action. Error bars show the 95% CI of the mean, determined over at least four replicates.

Consistent with oxidative stress contributing to growth impairment, all mutants showed increased protein carbonylation levels (Figure 4A), and treatment with the antioxidant N-acetylcysteine rescued the H_2_O_2_ sensitivity phenotype (Figure 3A). Further, we were able to reverse the phenotype by expressing wild-type copies of the deleted genes (average survival of 15 complemented mutants was 97.2% of wild-type, compared with 18.7% without the plasmid; see Additional file 9) indicating that the observed effect was not due to disrupted regulation of other genes or to background mutations in the deletion strain.

Finally, a literature search yielded additional evidence supporting a role in oxidative stress resistance for 4 of the 13 genes in *E. coli* or other organisms: *sufD*[28-30], *clpA*[31,32], and *gpmM*[33] in bacteria, and the orthologs of *icd*[34] and *gpmM*[35] in mice (see Additional file 10). Two additional genes, *lpd*[36] and *cysD*[37,38], are known to be targets of regulation during aerobiosis or oxidative stress in bacteria. Of the remaining seven genes, three have other known functions (*napF*, *rseC*, and *fre* are all oxidoreductases) while the other four genes (*yaaU*, *yidH*, *ybeQ*, *ybhJ*) are poorly characterized. The *fre* gene has a high-confidence predicted functional interaction with a catalase and a peroxiredoxin (see Additional file 11) in the STRING database [39], based on their correlated expression patterns, and the *cysD* gene to a thioredoxin reductase, based on text mining evidence (see Additional file 11). Interestingly, *ybhJ* is a catalytically inactive paralog of the *E. coli* aconitase enzyme [40], which is also known to act as a superoxide sensor and a regulator of stress response genes [41].

### The novel oxidative stress proteins contribute to homeostasis of iron and NAD(P)H

To better describe the specific roles played by these 13 proteins in oxidative stress defense, we measured cytoplasmic levels of reactive oxygen species (ROS) and found them to be increased compared with the wild-type in only 3 of the 13 strains: *cysD*, *rseC*, and *yaaU*; Figure 4A), as well as in the *sodA* positive control. Therefore, the majority of these genes appear not to act by detoxifying ROS, but instead by preventing or repairing the damage that ROS cause to cellular components.

Based on their known molecular functions (see Additional file 12) and the lack of increased ROS generation, we hypothesized two possible general roles for these novel genes in oxidative stress resistance: 1) that they function by maintaining the cellular redox state through supporting NAD(P)H production, and 2) that they influence iron homeostasis. These two roles are also suggested by the known functions of the predicted functional interaction partners of these novel genes as presented in the STRING database [39] (see Additional file 13). NAD(P)H levels are known to affect oxidative stress resistance in different ways, including the NADH-driven AhpC enzyme that detoxifies peroxides, or the NADPH-driven regeneration of glutaredoxins and thioredoxins, which reverse oxidative damage to proteins [42]. Iron is well known to aggravate the damaging effects of H_2_O_2_ through hydroxyl radical-generating reactions [43,44].

Given that oxidative stress is known to upregulate synthesis of NADPH at the expense of NADH in bacteria [45-47], we focused on the former metabolite. We found experimentally that 8 of the 13 deletion mutants did indeed have reduced NADPH levels (Figure 4A, B), including 3 that could be implicated in NADPH production from previous knowledge (*lpd*, *gpmM*, and *icd*; see Additional file 12) and 5 additional genes (*yaaU*, *cysD, rseC, ybhJ*, and *yidH*). Moreover, pre-treating the bacteria with exogenous NADPH rescued the H_2_O_2_-sensitive phenotype of all these strains, but none of the strains with normal NADPH levels, (Figure 4A, C), lending support to the hypothesis that the diminished NADPH levels of these eight strains are a contributing factor to the reduced H_2_O_2_ resistance.

To determine the gene products that might act via regulating iron levels, we measured total cellular iron, and found it to be at higher concentrations relative to the wild type in five of the deletion mutants (*fre*, *sufD*, *rseC, lpd*, and *yidH*; Figure 4A, 4B). Three of the deleted genes could be connected to iron-related processes based on previous knowledge (*fre*, *sufD*, and *rseC*; see Additional file 12). A complementary assay using the iron chelator 2,2′-dipyridyl showed that the *rseC*, *fre*, *sufD*, and *yidH* deletion mutants had diminished sensitivity to H_2_O_2_ after dipyridyl pre-treatment (Figure 4A, B), with some response noted for *lpd*. This outcome corroborated the putative role of these genes in helping maintain iron homeostasis.

While there are many proteins whose translation efficiency could have evolved as adaptation to oxidative stress, our experiments indicate that two important mechanisms that are actually employed are an abundant supply of biological reducing agents and careful management of iron levels.

### Validation of a role for codon optimality in additional phenotypic adaptations

**Figure 5:**
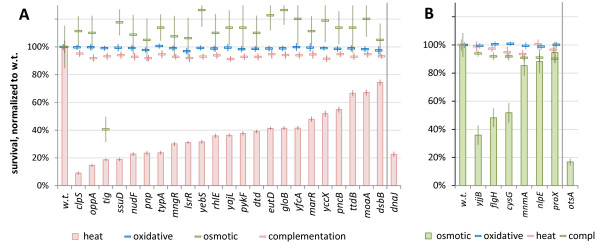
**Survival of *Escherichia coli *deletion mutants for genes in which codon adaptation was linked to thermophilicity or halophilicity.** Survival of strains with deletions in **(A)** putative heat stress and **(B)** putative osmotic stress genes discovered through differential codon adaptation in thermophilic or halophilic microbes (at false discovery rate (FDR) ≤6.6% and ≤33.9% respectively). Error bars show the 95% CI of the mean, determined over at least four replicates. Strains are ordered by survival, normalized to the wild-type survival under the same stress (23.6% for the heat and 21.3% for the osmotic shock after normalization, both shown as 100% on the plots). The *dnaJ* and *otsA* strains were included as a positive control.

To further investigate the generality of our methodology, we validated the predicted gene-phenotype links for two additional phenotypes: growth at increased temperatures and high salinity. Similarly to the H_2_O_2_ resistance experiments, we tested whether deletion of the orthologous *E. coli* gene from a COG with altered codon optimality in thermophile genomes proved deleterious after heat shock, while not affecting resistance to H_2_O_2_ and osmotic stress. Our experiments indicated a heat shock-specific protective role for 19 of 23 candidate genes (>40% decrease in mutant survival; Figure 5A), including the ClpS substrate modulator of the ClpAP chaperone-protease, which is known to direct its activity towards aggregated proteins [48]. Likewise, we also found that *E. coli* strains with deletions in three of six COGs with altered expression in halophiles had greatly decreased osmotic shock survival, but not decreased heat or H_2_O_2_ stress resistance (Figure 5B). The strongest response was seen in the mutant lacking *yjjB*, a conserved inner membrane protein of unknown function. For both thermotolerance and osmotic stress resistance, expressing the deleted genes from a plasmid reversed the phenotype of all mutants; average survival was 93.3% and 91.7% of the wild type for the 23 and 6 complemented mutants, respectively (compared with average 37.4% and 67.4% for the deletion mutants) (Figure 5A, B). Thus, just as for oxidative stress resistance, altered translation efficiency across species can be used successfully to identify new genes with other specific functions.

### Phenotypic effects of designed gene variants with reduced translation efficiency

While experiments on gene deletion strains can demonstrate the importance of a particular gene for a given phenotype, the natural changes in use of optimal codons are likely to cause less severe effects, such as changes in translation speed and/or accuracy. To show more directly that a change in the translation efficiency of the predicted gene families can bring about a phenotypic change, we selected two *E. coli* genes (*clpS* and *yjjB*) with prominent knockout effects on heat and osmotic shock survival, respectively (Figure 5A, B), and altered the genes’ codon usage.

**Figure 6:**
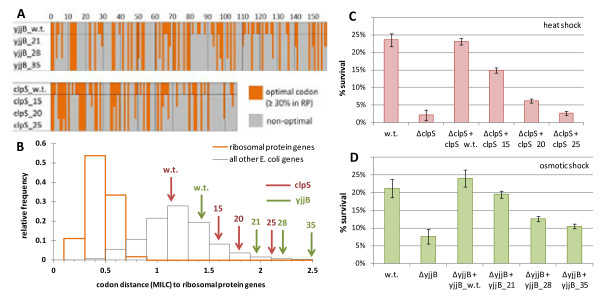
**Phenotypes of deletion mutants upon complementation with gene variants with different levels of codon adaptation. (A)** Design of *clpS* and *yjjB* gene variants with reduced proportion of optimal codons but with a preserved amino acid sequence. **(B)** Distributions of codon adaptation in *Escherichia coli* ribosomal protein genes (orange histogram) and other genes (grey histogram). The codon adaptation of the designed genes is marked by the arrows. **(C, D)** Heat and osmotic shock survival for the wild-type, the *clpS* and *yjjB* deletion mutants, and the same strains complemented by a wild-type or a gene variant with a reduced codon adaptation. Error bars show the 95% CI of the mean.

For each gene, we designed three sequence variants with unchanged protein sequence, but with progressively more optimal codons replaced by suboptimal ones (Figure 6A), thus tending toward the lower end of codon adaptation distribution in natural *E. coli* genes (Figure 6B) while still being within the range of observable codon usages. For all tested variants, the survival of *clpS* and *yjjB* deletion mutants complemented with the de-optimized genes was substantially lower than the survival of the wild-type *E. coli*, with a stronger reduction of survival in variants with a larger number of suboptimal codons. Expressing a wild-type gene fully rescued the heat/osmotic shock sensitive phenotype (Figure 6C-E). Of the other coding sequence features known to affect protein levels, secondary structures forming at the 5′ end of the mRNA are known to obstruct translation [25] if they are strong [7]. To rule out this variable, our sequence variants were designed to maximally conserve the original profiles of the mRNA folding free energies along the length of the genes (correlation with the wild-type *r ≥* 0.89 for all variants; see Additional file 14).

## Discussion and conclusions

The large number of sequenced prokaryotic and eukaryotic genomes presents an opportunity to better understand how organism physiology adapts to the environment. However, even in model organisms, a significant proportion of genes remains poorly functionally characterized [49]. Computational gene function inference methods can direct experimental work to discover the biological roles of such genes. One approach for predicting gene function is to use the presence/absence patterns of orthologous genes across species [50,51]. However, these ‘phylogenetic profiles’ capture only a subset of functional relationships [52], even though research efforts continue to gradually improve the methods for mining such data [53]. Similarly to the phylogenetic profiles, the signatures of gene expression levels reflected in codon biases are in principle discoverable in any organism for which the genome sequence is available. Such signatures are known to evolve in response to the environment within selected gene families and functional modules [9,12,14,16], and also to contribute to speciation in bacteria [54]. In addition, the environment has a global, genome-wide effect on codon biases; organisms adapted to living in multiple habitats exhibit a wider spread of codon usages between genes [55]. Here, we have described a method to systematically exploit the signal present in the synonymous sites of particular genes, while controlling for confounding variables such as phylogenetic proximity or correlated phenotypes. In this way, we were able to discern those correlations likely to correspond to true cause-and-effect relationships between the gene translation efficiency and the phenotype, providing a general scheme for prioritization of gene annotation experiments.

We would expect our predictions to be complementary to existing genome context methods [56]; for instance,, the functional interactions in STRING [39] predicted from gene occurrence in genomes, or the interactions from conserved gene neighborhoods, did not retrieve the same 34 oxidative stress COGs we found (no statistically significant difference from a random gene set; see Additional file 15). This implies that the predictions about oxidative stress genes that we have made and experimentally validated are not easily reachable by phylogenetic profiling or gene neighborhood methods. Interestingly, the set of discovered genes did not include the catalases *katE/G/P*, which are prominent *E. coli* antioxidant proteins. On closer inspection, the catalase COGs were enriched with HE genes in aerotolerant organisms (*katE* 3.4-fold, *katG/P* 3.2-fold), but they had very few gene representatives in aerointolerant microbes (see Additional file 16) and did not reach statistical significance. Therefore, catalases are an example of a gene family whose function is better discoverable by phylogenetic profiling [50], which correlates gene presence/absence with phenotypes (see Additional file 16; Fisher’s exact test *P* = 10^-25^ and *P* = 10^-14^ for *katE* and *katG/P*, respectively), serving to illustrate the complementary nature of the approaches.

To more systematically evaluate the sensitivity of our method, we performed a cross-validation test that retrieves *E. coli* genes with a known function through similarities of their translation efficiency profiles across different genomes (see Additional file 17). By extrapolation, we estimate that our method could retrieve on average 23% (10 to 29%; see Additional file 17) of the yet undiscovered genes relevant for different environmental responses. For comparison, phyletic profiling retrieves 32% (10 to 48%; see Additional file 17) of such unknown genes in the same setting. Therefore, the translation efficiency profiles possess around three-quarters of the detection power of the established phyletic profiling approach, but draw on an orthogonal and previously unexploited source of evolutionary signal. Moreover, the two methodologies can provide complementary gene function predictions: upon applying the models from the cross-validation test to a broader set of 3,534 *E. coli* genes, 69 genes were assigned a novel role in an environmental response exclusively by the translation efficiency profiles, while 86 genes were predicted a function exclusively by the phyletic profiles; 101 more genes had predictions by both methods (see Additional file 17).

We have experimentally demonstrated that genes exhibiting differential signatures of translational efficiency in aerobes and anaerobes have a role in defending *E. coli* from H_2_O_2_-induced oxidative stress. In addition to genes with previously unknown functions, we found genes with known roles in central metabolism to be important for averting oxidative damage, consistent with a known broad metabolic reprogramming of bacteria upon oxidative stress [57,58]. As the aerobicity-related changes in translation efficiency of these gene families across genomes could not be explained by the bacterial and archaeal phylogeny, the aerobes have likely independently evolved convergent codon bias patterns in different lineages under similar selective pressures. Our work, together with the aerobicity-related signatures in codon biases previously found in nine yeasts [16], will help describe the evolution of functional categories for surviving oxidative injury. Such findings may have implications for strategies to modulate the response of pathogens to macrophage assault, or, more broadly, for understanding ROS-induced pathologies and aging in higher organisms.

In addition to aerotolerance, we also predicted and experimentally validated the phenotypic consequences of the thermophilicity-related and halophilicity-related evolutionary codon bias signatures, thus further showing that our approach will be applicable to diverse phenotypes. Furthermore, for representative genes, we complemented the corresponding deletion mutants with synthetic variants that had altered codon optimality. Previous work used designed libraries of gene variants harboring synonymous mutations to demonstrate how they influence the levels of the corresponding protein [6,25]. Similarly, we experimentally changed the translation efficiency of selected genes, but in this instance we demonstrated an organism-level effect of the synonymous changes – a phenotypic change that recapitulates the discovered evolutionary trends. This validation of the pervasive gene codon adaptation-phenotype relationships that we found through computational analysis suggests that changes in translation efficiency may be broadly acting drivers of phenotypic change.

In summary, our work introduces a novel ‘genomic context’ approach to infer gene function from differences in translation efficiency. We anticipate that the power of this purely sequence-based methodology will grow as the number of fully sequenced genomes, as well as the systematic phenotypic annotations of organisms, increases. To facilitate further experimental work on other putative gene-phenotype connections, we supply the inferred 200 high-confidence links from all COGs to 24 phenotypes (see Additional file 18), as well as a broader set of thousands of predictions obtained at relaxed specificity thresholds.

## Materials and methods

### Analyzed genomes and predicted gene expression levels

We downloaded 1,275 fully sequenced prokaryotic genomes from the NCBI database [59]. Multiple strains of a single species were removed to counter biases toward commonly re-sequenced species, such as *E. coli* or *Streptococcus pyogenes*, resulting in 912 species-level representatives. Because we later used *E. coli* MG1655 as the experimental model to verify our predictions, its genome was removed from the set of analyzed genomes in order to avoid circularity, leaving a final set of 911 genomes; other *E. coli* strains were also removed.

In brief, the computational pipeline for predicting high/low expression of genes from the genome sequence (Figure 1A; see Additional file 1) involved training an RF classifier [60] to discriminate known HE genes (RPs and chaperones) from the rest of the genome using codon frequencies, and comparing the predictions of the trained RF model against those of a baseline RF model trained on composition of the neighboring intergenic DNA of these genes. This approach is a variant of the methodology presented by Supek *et al*. [9], therefore, we include a detailed description of the computational pipeline (see Additional file 1), as well as a list of the changes from the original version.

### Testing for correlation of lifestyles with gene expression

After obtaining the HE or non-HE label for each gene in the 911 genomes, we used Fisher’s exact test to call enrichment/depletion for HE genes within COGs in genomes grouped by environment, phenotype, or taxonomy. In particular, for each COG: 1) we tested whether its representatives are more/less frequently HE in 514 aerotolerant microbes compared with the 214 obligate anaerobes (note that some organisms had this phenotype undefined); 2) we checked within the aerotolerant microbes for enrichment/depletion of HE genes in 296 obligate aerobes in comparison to the 217 facultative aerobes; 3) we tested for enrichment/depletion of HE genes in 142 thermophiles versus 643 non-thermophiles; and 4) assessed enrichment/depletion of HE genes in 40 halophiles versus 140 non-halophiles (again, for the majority of organisms, the halophilicity was undefined).

Additionally, the same tests were performed for other descriptions of phenotypes or taxonomy, obtained as follows. Descriptions of the microbes’ environments or phenotypes were assembled from the NCBI Entrez Microbial Genome Properties website [61], followed by manual curation, particularly for pathogenicity phenotypes. All properties of interest were encoded as a series of binary (‘yes/no’ or ‘low/high’) categorical variables, possibly with missing values; the two continuous variables – GC content and genome size – were discretized into four classes. In total, this yielded 24 phenotypic features and 6 features describing the GC content and genome size (see Additional file 5). The organisms’ taxonomy was handled in a similar manner, where the possible taxonomic subdivisions at the domain, phylum, class and order level were encoded as ‘yes’/‘no’/‘not applicable’ categorical variables, yielding 70 features (see Additional file 5), for a total of 100 features per genome.

### Thresholds for COG size, enrichment, and statistical significance

We excluded from testing all COG groups with fewer than 20 representative genes in total (counted over all genomes, regardless of the phenotype), or with more than 10,000 genes in total, leaving 4,387 COGs of possible interest.

For each of these COGs, all phenotypes of interest were screened for enrichment with HE genes of two-fold or higher (or ≤0.5-fold depletion) in that specific phenotype. compared with the organisms known not to have the phenotype. These COGs were then tested for statistical significance of the enrichment using Fisher’s exact test (two-tailed) at *P* < 10^-2^. The four phenotypes that we subsequently validated experimentally were: 1) 514 aerotolerant microbes versus 214 obligate anaerobes: 295 of 2,847 tested COGs were significantly enriched/depleted for HE genes; FDR = 9.6%; 2) 296 obligate aerobes versus 217 facultative aerobes: 160 of 1,887 tested COGs were significantly enriched/depleted for HE genes; FDR = 11.8%; 3) 142 thermophiles versus 643 non-thermophiles: 346 of 2,287 tested COGs were significantly enriched/depleted for HE genes; FDR = 6.6%; and 4) 40 halophiles versus 140 non-halophiles: 55 COGs of 1,863 tested COGs were significantly enriched/depleted for HE genes; FDR = 33.9%.

### Controlling for confounding effects of other phenotypes and phylogenetic proximity

Even if a strong and highly significant correlation between increased expression in a COG and a phenotype is found, this in itself does not imply a causal relationship between the two variables. A common explanation involves the correlation being due to both variables being causally linked to a third variable (or to more variables). To control for such cases and prioritize the causal relationships within a potentially much larger number of correlations, we introduce a methodology based on supervised machine learning that measures whether a specific phenotype has an independent contribution to predicting gene expression levels, after controlling for all other phenotypes/environments and the phylogenetic relatedness. This computational method relies on the use of a classifier that can infer highly complex relationships involving many different independent variables (here: phenotypes or phylogeny) and one dependent categorical variable (here: the HE/non-HE labels on genes). In other words, the classifier ‘learns’ to predict gene expression for genes in a certain COG from the phenotypic, environmental, or phylogenetic relatedness of the corresponding organisms. The procedure consists of the following steps:

1) Construct the dataset. For each gene family (here, COG) make a separate dataset that has as many instances (examples) as there are genes in the COG (possibly >1 per genome), and as many independent variables (features) as there are phenotypes and phylogenetic subdivisions (here, 100), plus one dependent variable (class) with the predicted expression levels in the form of HE/non-HE labels.

2) Train the classifier and evaluate the model. Run the classifier and evaluate the accuracy of its predictions (here, using the area under the receiver operating characteristic curve (AUC) score [62]), while employing a cross-validation scheme. This setup penalizes overly complex models that over-fit to noise in the data, while rewarding models that generalize to unknown data better. Here, we used theRF [60] classifier as implemented in the FastRandomForest software [63] that integrates into the Weka Environment for Knowledge Analysis [64].

3) Repeat for randomized datasets. Shuffle a single dependent variable (here, phenotype) while leaving other phenotype/taxonomy-describing variables intact, and repeat the classifier training, and measure the cross-validation AUC score. Repeat this step 30 times while re-shuffling the same variable each time.

4) Test for consistent decrease in AUC score. Calculate a Z-score (number of standard deviations a measurement is away from the mean) for AUC_original_ compared with a distribution of 30 AUC_shuffled_ values. From the Z-score, find a one-tailed *P* value (using the cumulative distribution function of the normal distribution) that indicates whether the AUC score consistently decreases with randomization of the variable of interest.

5) For all phenotypes/environments of interest, repeat randomization test (steps 3 and 4). Here, these are the two tested aerobicity-related phenotypes; see section ‘Testing for correlation of lifestyles to gene expression’ above.

6) For all COGs, repeat steps 1 to 5.

The rationale behind the procedure is that shuffling one of the variables will destroy the information that variable might carry and that is relevant for predicting the high/low expression level. If this same information can be recovered from the other variables (possibly by combining them), the accuracy of classification will not be lowered by the randomization, whereas in cases where the variable in question is informative of the expression level of the genes in a way that cannot be substituted for by the remaining variables, the accuracy of the classification model will be reduced by randomization.

### Bacterial strains, growth conditions, and stresses

All the used strains as well as specifics of their construction are listed (see Additional file 19). All strains were derived from wild-type sequenced *E. coli* MG1655 by P1 transduction and/or transformation. Relevant plasmids were purchased from the ASKA library [65]. Bacteria were grown in LB at 37°C, to the mid-exponential phase (OD_600_ = 0.2 to 0.3).

For the H_2_O_2_ treatment, they were washed in 0.01 M MgSO_4_ and incubated at 37°C for 20 minutes in the absence and presence of 0.5 mM, 2.5 mM, and 20 mM H_2_O_2_. Osmotic shock was achieved by exposing exponentially growing *E. coli* to 1 M NaCl (final) for 1 hour, and heat shock was achieved by growing *E. coli* at 56°C for 100 minutes. Viable cell counts were always estimated by plating serial dilutions on LB plates and growing overnight at 37°C.

To test if the mortality of *E. coli* deletion mutants upon exposure to H_2_O_2_ was caused by the increased ROS production, we performed a rescue experiment using 5 mM N-acetyl cysteine (NAC), a known ROS scavenger. Overnight cultures of *E. coli* were diluted 200 times and grown in the presence ofthe 5 mM NAC until the mid-exponential phase. Cells were then washed and oxidized with 2.5 mM H_2_O_2_ for 20 minutes, and survival was measured as described above.

### Measuring protein carbonylation and reactive oxygen species production

Exponentially growing bacteria were harvested from LB medium. *E. coli* cells were pelleted by centrifugation and resuspended in 10 mM PBS (pH 7.4), supplemented with a mixture of protease inhibitors (Roche, Basel, Switzerland). Resuspended cells were frozen immediately in liquid nitrogen. Cells were broken by a mechanical homogenizer, and centrifuged for 20 minutes at 12,000 × g. Samples were then supplemented with 10 mg/100 μl lipid removal agent (13360-U; Sigma, St. Louis, Missouri, USA), incubated for 1 hour at room temperature with shaking, and centrifuged for 15 minutes at 10,000 × g. The amount of protein in the supernatant was measured by the Lowry method. Protein extracts diluted to 10 μg/ml were loaded into wells (Maxisorb; Nunc, Roskilde, Denmark) and incubated overnight at 4°C to allow proteins to adsorb to the surface, followed by 0.6 mM dinitrophenyl hydrazine (DNPH) derivatization of adsorbed proteins and detection of derivatized dinitrophenol (DNP)-carbonyl by a mouse DNP-specific monoclonal antibody conjugated to horseradish peroxidase. Subsequent incubation with enzyme substrate 3,3′,5,5′-tetramethylbenzidine (TMB; Sigma, St. Louis, Missouri, USA) resulted in a colored product that was quantified using a microplate reader at 450 nm.

ROS levels were determined by labeling *E. coli* strains with 25 μM dihydrorhodamine-123 for 10 minutes in the dark, in the absence or presence of hydrogen peroxide. Cells (approximately 10^6^) were then washed in minimal medium, and their fluorescence was measured with excitation at 500 nm and emission at 530 nm. In addition, *E. coli* strains were labeled with CellROX™ Deep Red reagent (Invitrogen, Carlsbad, California, USA) in the absence or presence of hydrogen peroxide. Cells (approximately 10^6^) were washed in minimal medium, and their fluorescence was measured with excitation at 630 nm and emission at 665 nm.

### Measurement of cellular NADPH and Fe, and rescue experiments

We measured intracellular NADPH level using a commercial kit (Vybrant Cytotoxicity Assay Kit; Molecular Probes, Eugene, Oregon, USA) that is normally used to monitor the release of the enzyme glucose 6-phosphate dehydrogenase (G6PD) from damaged cells. Oxidation of glucose-6-phosphate by G6PD results in the generation of NADPH, which in turn leads to the reduction of resazurin by diaphorase to yield fluorescent resorufin. We took advantage of the second reaction to measure NADPH levels directly, while filtering the cellular extract of each studied strain through a 3 kDa cutoff centrifugal filter (Amicon Ultra; Millipore, Billerica, Massachusets, USA) to prevent the cellular proteins (including G6PD) from creating a background with the reaction mixture. A sample (100 μL) of each cellular filtrate was distributed into wells twice in duplicate, and the level of NADPH was determined as follows. A reaction mixture was prepared by dissolving a lyophilized mixture of diaphorase, glucose-6-phosphate, and NADP^+^ (Component C of the kit) in 0.5 M Tris buffer pH 7.5 (Component D of the kit). The reaction mixture was then combined with the solution of resazurin so that the final concentration of resazurin was 30 μM (component A). Then, 100 μL of the final mixture was loaded onto the samples distributed in the wells, and incubated at 37°C for 5 hours. The amount of NADPH was measured as the absorbance at 570 nm.

To test which *E. coli* strains were rescued by pre-treatment with NADPH, exponentially growing *E. coli* strains were first exposed to 1% v/v toluene in the presence of 10 mM EDTA (known to permeabilize the bacterial membranes to NADPH [66]) and then exposed to 20 μM NADPH dissolved in 10 mM PBS, (pH 7.4). Cells were then treated with H_2_O_2_, washed in 0.01 M MgSO_4_, and incubated at 37°C for 20 minutes in the absence or presence of 2.5 mM H_2_O_2_. Viable cell counts were estimated by plating serial dilutions on LB plates and growing overnight at 37°C.

We measured the level of cellular iron (both Fe^2+^ and Fe^3+^) as described by Rad *et al*. [67]. In particular, about 10^7^ exponentially growing *E. coli* cells were pelleted and incubated overnight at 110°C without tube caps. After evaporation of liquid, 1 ml of 10 M HCl was added, and samples were incubated for 4 h at 60°C. Next, the content of each tube was diluted twofold with 10 M HCl, and absorbance was measured at 351 nm. To test which *E. coli* strains were rescued by pre-treatment with 2,2′-dipyridyl (iron chelator), exponentially growing *E. coli* strains were exposed to 0.4 mg/ml (final concentration) dipyridyl. Cells were then treated with H_2_O_2_ as described above, and viable cell counts were estimated by plating serial dilutions on LB plates and growing overnight at 37°C.

### Phenotypic effects of introducing synonymous changes in the *clpS* and *yjjB* genes

For each of the two selected *E. coli* genes, we designed three additional variants with synonymous changes: for *clpS*, 15, 20 and 25 optimal codons were replaced with non-optimal ones, and for *yjjB*, 21, 28, and 35 codons were changed. The number of changed codons was chosen to be proportional to the sequence length (*clpS* is 107 codons long and *yjjB* is 158 codons long). The optimality of a codon was defined as its frequency in the *E. coli* RP)genes, normalized to sum to 100% for each amino acid. All introduced changes had to reduce the optimality of the original codon by at least 30% below the original value, while not falling below 3% to avoid the extremely rare codons such as the AGG or AGA arginine codons (0.6% and 0% usage in *E. coli* RP). Therefore, with our gene variants, we aimed to incorporate a large number of moderate changes in codon optimality, rather than a small number of drastic changes, assuring a more even distribution of the codon optimality levels along the length of the gene. In the *yjjB* sequences (including wild-type sequence), we also abolished a HsdR site, AACGTTCCCGTGC, by changing CCC-GT**G**-C to CCC-GT**A**-C (a synonymous change, where one suboptimal valine codon was exchanged for another).

To control for stable secondary structures in the mRNA that may inhibit protein translation independently of the use of optimal codons, we used a script that in each step replaces five (for *clpS*) or seven (for *yjjB*) randomly chosen codons in the sequences with suboptimal ones (while obeying the rules described above), repeating the random selection 100 times, and selecting the variant with the predicted RNA folding energy profile most similar to the original gene. Then, another set of five or seven codons are replaced, again with 100 random samplings, keeping the least changed RNA folding profile, and so on. The RNA folding free energy profiles for the genes were calculated for the 42-nucleotide folding windows using the *hybrid-ss-min* program from the UNAFold 3.6 package [68], with default parameters (NA = RNA, t = 37, [Na+] = 1, [Mg++] = 0, maxloop = 30, prefilter = 2/2). The difference in RNA folding profile between the mutated and the original sequence was computed as the root mean square deviation of folding free energies for all 42-nt windows. All sequences are given in Additional file 20, and the RNA secondary structure folding free energy profiles are given in Additional file 14.

The *clpS* and *yjjB* deletion mutants were complemented with a pJ801 plasmid encoding either the wild-type gene, or the variants with introduced synonymous mutations described above. The plasmids with the appropriate inserts were purchased from DNA2.0 and bore a kanamycine resistance cassette, and the genes were under the control of a rhamnose-inducible promoter. Overnight cultures of *E. coli* strains were diluted 200 times in LB medium, supplemented with 1.5 μM rhamnose and grown for 2 to 3 hours to an OD of 0.2 to 0.3. The *clpS* mutants were exposed to heat shock (100 minutes at 56°C) and *yjjB* mutants to osmotic shock (1 hour at 1 M NaCl and 37°C) and survival measured as for the deletion mutants.

## Competing interests

The authors declare that they have no competing interests.

## Authors’ contributions

AK and TC carried out all experimental assays; FS conceived the study, performed the computational analyses, and drafted the manuscript; and AK, TG, and BL participated in the study design and data interpretation, and contributed substantially to the manuscript. All authors read and approved the final manuscript.

## Supplementary Material

Additional file 1 **Supplementary Materials and Methods.** Contains sections on: creating reference sets of highly expressed genes; the random forest classifier; detecting selection for translational efficiency in genomes; assigning ‘highly expressed’ labels to individual genes; a figure with a schematic of the computational workflow; comparison with the procedure from Supek *et al*. [9]; gene expression data sources; and normalization of experimental results. Click here for file

Additional file 2 **Agreement with expression data for the predictions about highly expressed (HE) genes, and a comparison with the original ‘optimized codon usage’ (OCU) method [**[9]**].***P* values are from a Mann-Whitney test for a difference in microarray signal levels between the HE and non-HE genes, or the OCU and non-OCU genes. The ‘ratios’ were calculated between the average microarray signal of the two groups. The ratio of ribosomal proteins versus whole genome is given for a sense of scale; the ribosomal protein genes are expected to be very highly expressed. Click here for file

Additional file 3 **The relative proportion of highly expressed genes is lower in larger genomes.** This correlation was previously explained [9] by different proportions of various gene functional categories in smaller or larger genomes. Many of the functional categories, in turn, tend to have a general preference for higher or lower expression. For instance, larger genomes have a disproportionally increased number of gene regulators, which have a strong tendency to low expression. Smaller genomes, on the other hand, have a higher proportion of ribosomal proteins, whose absolute number is roughly fixed across genomes, regardless of their size. Click here for file

Additional file 4 **Correlations of mRNA 5′ end folding free energies and various codon indices with gene expression levels.** The free energies are a measure of the stability of the structures (more negative = more stable) and are calculated in windows of 42 nucleotides in length on the mRNA sequence using the *hybrid-ss-min* program from UNAFold 3.6 with default parameters, as in [25]. The three 42-nt window positions investigated are: (-4 to 37), found to have a strongest correlation to protein levels [25]; (-20 to 21), a window centered over the start codon; and (-30 to 11), a window centered on the common location of the Shine-Dalgarno sequence at -9 [69]. The -10 kcal/mol figure is the approximate limit for the mRNA folding free energy in 42-nt windows; at negative values below this, the mRNA folding starts to have a considerable effect on translation efficiency [7]. The mRNA coordinates are given relative to the start codon, where 1 is the A in AUG. The codon indices are: CAI [4], B [70], and MILC [5]. RF, probability score obtained from a random forest classifier [9]. All codon indices use the same ‘reference set’ of known highly expressed genes as used in our analyses (see Supplementary Methods in Additional file 1). Click here for file

Additional file 5 **The 100 features describing each organism which were used in the search for the phenotypes predictive of the changes in translation efficiency within clusters of orthologous groups (COGs).** All features are binary variables, and can be undefined for some organisms. We included 70 features describing the phylogeny (left/middle columns) and the 6 features describing genome size and GC content (right column, top) to ensure that correlations detected with the remaining 24 features (phenotypes, right column) could not be explained by the phylogeny or the genomic size/GC. #pos, number of organisms marked as positive for a specific feature; #neg, number of organisms marked as negative for a specific feature. Click here for file

Additional file 6 **Genomes for which the optimal codons inferred from over-representation in highly expressed (HE) genes overall did not match the expected optimal codons inferred from the genomic tRNA repertoire.** The nine twofold degenerate amino acids were examined. An optimal codon (HE column) was defined as over-represented at *P* < 0.001 in a Fisher’s exact test on codon counts in HE versus the non-HE genes; a non-significant result means no codon is optimal. The codons expected to be optimal from tRNAs tRNA column) are defined in the genomes in which tRNA genes with only one of the two possible anticodons were present (found by tRNAscan-SE), then the codon matching that anticodon by canonical Watson-Crick pairing was considered tRNA-optimal, and the other codon, which uses wobble pairing, was considered tRNA-suboptimal. The table shows 71 (of the 911 total) genomes for which the optimal and the tRNA-optimal codons disagreed in at least 3 of 9of the testable amino acids (# aa column). In 651/911 genomes, there were 0/9 disagreeing amino acids, and 1/9 for a further 135 genomes. Thus, in the 71 genomes, the expression level-related codon bias did not, overall, clearly relate to the tRNA gene repertoire, and may possibly not reflect translational selection, but rather another, unknown factor. We thus excluded the 71 genomes, and re-ran the subsequent analyses to verify if our findings were robust to inclusion of these genomes (see Additional file 7). Click here for file

Additional file 7 **Robustness of the 200 discovered clusters of orthologous groups (COGs)-phenotype links to the exclusion of 71 genomes for which codon biases were not clearly related to the tRNA gene repertoires.** Excluded genomes are listed in Additional file 6. **(A)** The log_2_ enrichment of the 200 COG-phenotype links with the full set of 911 genomes, and after exclusion of the 71 genomes. **(B)** Same as **(A)**, but limited to the links that we experimentally validated. In the original analysis, a threshold of log_2_ enrichment of ≥1 or ≤-1 was a requirement for calling the 200 COG-phenotype links; after excluding the 71 genomes, 195 COG-phenotype links still met this criterion. **(C)** The log_10_*P* value for significance of the enrichment/depletion (two-tailed Fisher’s exact test), again compared between the original and the reduced genome sets. **(D)** Same as **(C)**, but only for the COG-phenotype links with log_10_*P*≥-6. In the original analysis, log_10_*P*≤-2 was required for calling the 200 COG-phenotype links; after excluding the 71 genomes, 173/200 links still had log_10_*P*≤-2, and 185/200 still had log_10_*P*≤-1.7 (*P* < 0.02). Click here for file

Additional file 8 **Survival of *Escherichia coli *deletion mutants after oxidative stress induced by different hydrogen peroxide concentrations.** Survival after heat and osmotic shock is given for comparison. Deleted genes are on the *x* axis. The *y* axis shows the survival of the mutant, normalized to the survival of the wild type *(*w.t.) under the same conditions, which was 45.6% for 0.5 mM H_2_O_2_, 13.8% for 2.5 mM H_2_O_2_, 4.2% for 20 mM H_2_O_2_, 23.6% for heat shock, and 21.3% for osmotic shock. The *lon* and *recA* mutants are shown separately as they exhibited a non-specific stress response, being sensitive also to osmotic and heat stress. *sodA* is a known oxidative stress defense gene, serving as a positive control. Click here for file

Additional file 9 **Complementing *Escherichia coli *deletion mutants with wild-type genes.** Survival of *E. coli* deletion mutants in the putative oxidative stress response genes with and without the corresponding genes expressed from a plasmid. Click here for file

Additional file 10 **Supporting evidence for putative oxidative stress genes.** A survey of the evidence in the literature offering support for the involvement of *sufD*, *clpA*, *icd*, *gpmM*, *lpd*, and *cysD* genes in oxidative stress resistance of various organisms. Click here for file

Additional file 11 **Functional interactions with known oxidative stress genes.** Predicted functional interactions between 34 clusters of orthologous groups (COGs) we found to have codon adaptation that correlates with the aerobic lifestyle, and 30 COGs encoding known *Escherichia coli* oxidative stress response proteins. The predicted interactions are from the STRING v9.0 database, using exclusively co-expression (top part of table), or exclusively text mining (bottom part) evidence. Only interactions marked as high confidence by STRING (confidence ≥0.7) are shown. Click here for file

Additional file 12 **Literature data suggesting putative antioxidant mechanism of action assignments.** Listed for the *sufD*, *fre*, *rseC*, *gpmM*, *lpd*, and *icd* genes. Click here for file

Additional file 13 **The functional context of the 13 *Escherichia coli *gene representatives of the clusters of orthologous groups (COGs) differentially expressed in aerobic microbes.** The genes *recA* and *lon* are not shown because their deletion mutants showed non-specific stress sensitivity (Figure 3). Lines represent the predicted functional interactions from the STRING 9.0 database (medium confidence level, ≥0.4), while dots represent all proteins interacting with at least 1 of the 13 proteins. A large, highly interconnected set of interacting ribosomal proteins is not shown for clarity. The larger, colored dots are proteins annotated with one of the selected functional categories in *E. coli* (right panel). Hollow circles in *fre* or *rseC* or thick border in *napF* denote putative assignments we inferred for these genes from the literature; all other functional annotations were from the Uniprot-GOA (Gene Ontology Annotation) database. All shown functional categories were found to be enriched among the 13 proteins plus interactors at *P* < 0.05 (hypergeometric distribution, corrected for multiple testing) using GeneCodis 2.0. Proximity of the circles in the figure roughly corresponds to their functional similarity, as optimized by the Edge Weighted Spring Embedded layout in Cytoscape 2.8.1, edge weights being derived from interaction confidence levels in STRING. Click here for file

Additional file 14 **Profiles of folding free energies in 42-nucleotide windows along the *clpS *and *yjjB *gene mRNAs.** The *x* axes show the starting coordinate (in nucleotides) of the 42-nt window. The folding free energies were calculated using the *hybrid-ss-min* program from UNAFold 3.6 software with default parameters. Alongside each *Escherichia coli* gene (marked ‘w.t.’), three variants are given with introduced synonymous changes that reduce codon optimality (Figure 5); the number given after the word ‘variant’ is the number of codons that have been altered with respect to the wild type. A 14-nt ribosome binding site sequence, AGGAGGUAAAACAU, was prepended before the AUG start codon when determining the folding free energies, as was the case for the actual genes. For each variant, Pearson’s correlation coefficient, *r*, and the root mean square deviation (RMSD) are given as measures of similarities of their folding free energy profiles to the wild-type sequence. Click here for file

Additional file 15 **Distributions of predicted functional interactions at different confidence levels.** Functional interactions were examined between (1) 30 clusters of orthologous groups (COGs) known to have a role in the oxidative stress response, labeled ‘known versus known’; (2) the ‘known’ group and the 34 COGs found to be differentially expressed between aerotolerant organisms and anaerobes, or between obligate and facultative aerobes, labeled ‘diffExpr versus known’; and (3) the ‘known’ group and a 100 randomly chosen COGs, labeled ‘randomSet versus known’. Two of the 34 COGs were also in the ‘known’ group, and their functional interactions did not count for the ‘diffExpr’ group; these were COG0719 (*E. coli sufD* and *sufB* genes) and COG1249 (*E. coli lpd*, *ykgC*, *gor*, and *sthA* genes). The predicted functional interactions are from the STRING v9.0 database [39], the scores vary from 0 to 1; STRING declares interactions between 0.15 and 0.40 to have low confidence, between 0.40 and 0.70 to have medium confidence, and above 0.70 to have high confidence. For details on how the scores are computed for each individual source of data, please refer to references given at the STRING website. The *P* values are from a χ^2^ test. Click here for file

Additional file 16 **Relationships of the aerotolerance phenotype with the presence/absence patterns and with the codon adaptation of the catalase genes.** Tables show the count of organisms (not genes) with the clusters of orthologous groups (COGs) being absent (first column), present with one or more genes that are all non-highly expressed (HE) (second column), or present with one or more genes of which at least one in the genome is HE (third column). The tables below show the same frequencies, but normalized to the total number of aerotolerant or strictly anaerobic organisms. For both COGs, the presence of the catalases in the genome is strongly and significantly correlated with aerobicity (top right panel for each COG). However, the codon adaptation of the catalases is strongly but not significantly correlated with aerobicity (bottom right panel for each COG), because of the low numbers of strictly anaerobic genomes that have a catalase gene present. Click here for file

Additional file 17 **A cross-validation test of the ability to retrieve functionally related genes, starting from the translational efficiency profiles of clusters of orthologous groups (COGs) across genomes (left panel), or the gene presence/absence profiles (right panel, equivalent to a standard phyletic profiling approach).** The test uses *Escherichia coli* K12 genes that are assigned to a COG and that are annotated with one of the five Gene Ontology (GO) categories above, and compares these genes with a sample of other *E. coli* genes that are in COGs but that do not have this GO function assigned. The size of the sample of these ‘negative genes’ is 19 times the number of ‘positive’ genes, which thus make up 5% of the combined dataset, mimicking a realistic distribution. Next, a Random Forest model is trained to discriminate the two groups of *E. coli* genes, and tested in a *n*-fold cross-validation scheme (using Weka 3.7.9), where *n* is the number of positive genes for that GO. The plots are precision-recall curves: recall is on the *x* axis, precision on the *y* axis. Importantly, the translation efficiency models (left panel) do not have access to gene presence/absence information, and must discriminate the groups only from the codon biases of the present genes; absent genes are encoded as missing data. The measure of translation efficiency in the profiles is the difference in classifier probabilities of the intergenic DNA versus codon usage data (Figure 1A, left versus right). Click here for file

Additional file 18 An exhaustive list of the inferred clusters of orthologous groups (COGs)-phenotype links. Click here for file

Additional file 19 **A list of *Escherichia coli *** **strains used.** Click here for file

Additional file 20 **Designed variants of *Escherichia coli clpS *and *yjjB *genes, with progressively more optimal codons replaced by suboptimal ones (Figure** 6**).** The lowercase ‘a’ in the *yjjB* sequences denotes a replacement of the original G with an A to abolish a HsdR site. Click here for file
