# Supplementary material for: Inferring gene function from evolutionary change in signatures of translation efficiency
Source: Genome Biol. 2014 Mar 3;15(3):R44. doi: 10.1186/gb-2014-15-3-r44 (PMC4054840; doi:10.1186/gb-2014-15-3-r44)
Supplement: Additional file 1 — Supplementary Materials and Methods. Contains sections on: creating reference sets of highly expressed genes; the random forest classifier; detecting selection for translational efficiency in genomes; assigning ‘highly expressed’ labels to individual genes; a figure with a schematic of the computational workflow; comparison with the procedure from Supek et al. [9]; gene expression data sources; and normalization of experimental results. [file gb-2014-15-3-r44-S1.docx]

## Supplementary Methods for Kriško *et al*.

### Reference sets of highly expressed genes

In each genome, we declare all genes coding for ribosomal proteins to be the ‘reference set’, assuming them to be very highly expressed, therefore exhibiting a strong influence of translational selection on codon usage. In addition, some other translation-related genes are included in the reference set:

- translation initiation factors:
  - in Bacteria, COGs 0361 (IF-1), 0532 (IF-2) and 0290 (IF-3);
  - in Archaea, COGs 0023 (eIF-1), 1093 (eIF-2α), 1601 (eIF-2β), 5257 (eIF-2γ)
- translation elongation factors:
  - COG 0480 (EF-G), 0264 (EF-Ts), 0050 (EF-Tu), 0231 (EF-P), 2092 (EF-1β), 5256 (EF-1α)
- chaperones:
  - COG 0459 (GroEL / HSP60), 0234 (GroES / HSP10), 0443 (HSP70), 0484 (HSP40), 0576 (GrpE), 0544 (trigger factor), 1832 (prefoldin)

Genes shorter than 80 codons, having internal stop codons or having length (in nucleotides) indivisible by three were excluded from computation; this includes some of the 'reference set' genes. A gene is then represented by a series of codon frequencies for all degenerate codon families, excluding the stop codons and the rare amino acids cysteine and histidine. The frequencies of codons for a single amino acid are adjusted to sum to one, therefore the final data does not reflect amino acid frequencies. If an amino acid is absent from a gene product, the amino acid’s codon frequencies are not estimated in any way but are instead represented by a special ‘missing value’ symbol.

### The Random Forest classifier

We employ a supervised machine learning method – the Random Forest (RF) classifier (Breiman 2001) – to detect signatures of selection acting to optimize translational efficiency of genes. The RF implementation we use is FastRandomForest 0.98 (Supek 2011) which integrates into the Weka learning environment (Witten and Frank 2005). RF was our method of choice as it is computationally efficient, robust to noise and handles missing data. The 'forest size' parameter ("-I") was set to 1000; other parameters were left at default values.

The RF algorithm produces an ensemble of decision tree classifiers, where each decision tree is constructed by recursively partitioning the data by attribute value tests (forming ‘nodes’) so as to reduce the class entropy in the resulting partitions (‘branches’). In RF, trees are constructed on bootstrap samples of the entire dataset, and choice of attributes at each node is restricted to introduce variability. The final predictions of a RF model are obtained by averaging over individual trees (‘voting’).

### Detecting selection for translational efficiency in genomes

Nucleotide substitution patterns in DNA molecules influence codon frequencies of genes; additionally, the biases in nucleotide substitution patterns may be region, strand, or chromosome specific (Daubin and Perriere 2003, Rocha 2004). Thus, it is necessary to control for the biases in order to reliably detect influence of translational selection on codon biases.

We encode the information about local nucleotide substitution patterns affecting each gene by computing mononucleotide and dinucleotide frequencies in the non-coding regions of DNA neighboring the coding region of the gene. Genes for known functional RNA molecules such as tRNA and rRNA are also treated as coding regions and thus do not contribute toward mono- and di-nucleotide frequencies of intergenic DNA. Additionally, the first 20 nt upstream of each coding region were masked and not taken into consideration when sampling the ncDNA, as this region region of prokaryotic ncDNA was found to be under much stronger selective pressures than the rest of the ncDNA (Molina and van Nimwegen 2008). First 50 available (not masked) non-coding nucleotides upstream of the start codon, together with the first 50 available nucleotides following the stop codon were used as the ncDNA sample for each gene. We also tested wider ±100 and ±200 nt intervals; see below.

To verify that translational selection acts in each genome - a prerequisite for predicting gene expression from codon usage patterns - we employ the following procedure: the RF classifier is first trained to distinguish the reference set genes based on the mono- and di-nucleotide frequencies of genes’ neighboring non-coding DNA; the codon frequencies are also supplied to the classifier at this point, but are shuffled between genes, thus rendered uninformative. 100 runs of four-fold crossvalidation are used to estimate the accuracy of the classifier using the area-under-ROC-curve (AUC) (Fawcett 2006) score, and the AUC for each of the 100 runs of crossvalidation is recorded. The codon frequencies are re-shuffled between genes in each run.

The procedure is then repeated for a second time, however now the true (unshuffled) codon frequencies are included in the dataset for the RF classifier training. The AUC scores are again recorded for each of the 100 runs of crossvalidation. To determine if selection for translational efficiency acts on the genome as a whole, the sign test (McDonald 2008) is used to compare 100 AUC scores obtained with shuffled codon frequencies to 100 AUC scores obtained with true codon frequencies, for each genome. If the AUC score exhibits a consistent increase over 100 runs of crossvalidation, the introduction of codon frequencies improves the ability to discriminate the reference set genes, providing evidence that translational selection acts on this specific genome. This was the case at *P*≤10^-25^ (sign test), corresponding to FDR≤9·10^-23^, for all but one of the genomes, Candidatus*Hodgkinia cicadicola Dsem* , a highly reduced genome (<200 genes) with high GC content (McCutcheon and Moran 2009) ; here, the difference of AUC scores was still significant at *P*≤10^-4^(FDR=9.1%). Such a result agrees with the previous findings about selected codon biases being universal among microbial genomes (Supek et al. 2010, Hershberg and Petrov 2009), indicating it is feasible to predict gene expression levels from codon usage in all of the analyzed genomes.

### Assigning 'highly expressed' labels to individual genes

In order to check whether each specific gene is affected by translational selection, during the procedure described above which involves two rounds of RF classifier training, the per-gene probabilities of belonging to the 'reference set' are recorded for each of the 100 runs of crossvalidation. The per-gene probabilities are then compared between the two rounds of crossvalidation - the round with the shuffled vs. with the intact codon frequencies. A sign test (McDonald 2008) is used to determine if an increase in probability occurs more frequently than expected by chance; if it does, the gene is labeled as highly expressed. The Supplementary Figure SM1 provides a schematic overview of the computational pipeline.

**Supplementary Methods Figure SM1.** A schematic describing the computational workflow employed to determine highly expressed (HE) genes in genomes.

We have set the sign test threshold *P* value to 10^-15^; this *P* corresponds to at least 88 out of 100 sign test ‘wins’ for the dataset with intact codon frequencies. Given that all genomes analyzed had <10^4^ genes, the used P value threshold corresponds to <10^-11^ false positive HE genes per genome. Also, given the smallest number of HE genes per genome was 13 (for the *Hodgkinia* above), the highest possible FDR for detecting the HE genes using the sign test is 10^-11^/13 ≈ 10^-12^.

This sign test *P* value should be regarded as somewhat liberal because the repeated runs of crossvalidation are not independent, being based on repeated sampling from the same set of genes. To obtain an additional, more conservative estimate, we employed a corrected paired *t*-test (Nadeau and Bengio 2003) originally intended for comparison of classification algorithms using repeated runs of crossvalidation. Note that we actually compare RF models derived from a specific dataset, and not the different variants of the underlying RF algorithm itself, and therefore this *P* value likely is pessimistic for our experimental setup (Nadeau and Bengio 2003). For example, using this very conservative *t*-test, the median *P* value for highly expressed genes in the *E. coli* K-12 genome would be 5.6·10^-4^, while for 95% of the HE genes, *P*<0.04*.* For the *Bacillus subtilis 168* genome, the median of this conservative *P* value estimate for HE genes would be 5.6·10^-4^, while for 95% of the HE genes *P*<0.07.

The assignment of the HE/non-HE label to genes depends on a comparison – via the described statistical test – of a gene’s codon usage to the dinucleotide composition of neighboring non-coding DNA. As stated above, our analyses were performed with 50 upstream + 50 downstream nucleotides per gene (total 100nt). We also evaluated whether taking neighborhoods of different sizes might have a bearing on the outcome by examining the agreement of the HE labels to experimental gene expression data from NCBI GEO in 19 genomes (sources listed in section below). The results are summarized in the Figure SM2 below: taking wider intervals does not consistently improves either the ratio of average HE/non-HE expression, or the *P* value of a Mann-Whitney test for separation in expression levels between the HE and non-HE groups.

**Supplementary Methods Figure SM2.** Agreement of the HE/non-HE labels to gene expression data in 19 bacterial genomes, depending on the width of the non-coding DNA neighborhood that is used as a baseline. Order of the genomes same as in Fig 1C.

### A comparison to the procedure from Supek *et al.* (PLOS Genetics 2010)

Note that the procedure described above is a derivative of the computational workflow described in reference (Supek et al. 2010) with several changes that resulted in better agreement to microarray measurements of gene expression in 19 phylogenetically diverse bacteria; see below for the list of microarray datasets. The average ratio of expression between the highly expressed genes and the rest of the genome has increased from 2.4x to 3.9x; the *P* value for separation of the expression value in the two classes has improved from 2·10^-12^ to 7·10^-48^ (Mann-Whitney test, median over 19 genomes); see Table S1 for the full data. The changes to the algorithm encompass:

- The original algorithm (Supek et al. 2010) uses 50 runs of 4-fold crossvalidation per genome; here, 100 runs of 4-fold crossvalidation are used. This requires more computational time but decreases the variability between repetitions of the pipeline.
- Originally, the first (of the two) round of crossvalidation uses just mono- and di-nucleotide frequencies, while the second uses both these, and the codon frequencies; here, both sources of data are present in both rounds, but codon frequencies are shuffled in the first round. This ensures that all comparisons are made between classification models with an equal number of degrees of freedom. The datasets have 75 features: this includes 55 codon frequencies (64, minus 3 stop codons, 1 Met, 1 Trp, 2 Cys and 2 His codons), 16 dinucleotide and 4 mononucleotide frequencies.
- The original reference set contained only ribosomal protein genes; here, the set is expanded using translation factors and chaperones (see above). These genes were typically used as parts of reference sets in previous studies, see eg. (Karlin and Mrazek 2000), as they are considered to have strong translational codon usage biases in various genomes.
- Originally, all ncDNA in a fixed-size window around each gene is collected (10 kb upstream of the start and 10 kb downstream of the stop codon); here, first 50 non-coding nt upstream of the start and first 50 non-coding nt downstream of the stop are collected, regardless of the distance. This ensures each gene is represented with an equal quantity of ncDNA.
- Originally, all amino acids were considered. Here, the rare amino acids cysteine and histidine are excluded as they were frequently present in very few instances or completely absent from a gene, introducing noise.
- Here, the Random Forest classifier is configured to put a heavier weight on the minority class (this is always the reference set), equal to 1/proportion of reference set genes. Originally, the two classes were treated with equal weights, despite the large imbalance in their sizes.

### Gene expression data sources

The datasets with absolute mRNA abundances in prokaryotes were downloaded from the NCBI Gene Expression Omnibus (GEO). The mRNA abundance data was obtained either using single-channel microarrays (commonly Affymetrix) or two-channel microarrays normalized to genomic DNA. We have manually curated to list to include only datasets where growth conditions can be expected to lead to fast growth of the organism, i.e. the conditions where translational selection would be expected to act – exponential growth phase, rich medium, and no physical, chemical or biological stress for the organism. Depending on the growth conditions, the correlation of gene expression to codon usage might be larger or smaller. Finally, we have reduced the list to one dataset per organism, leaving us with 19 datasets with good coverage of various bacteria phyla: Proteobacteria (α, ß, γ and δ), Firmicutes, Actinobacteria and the Thermus/Deinococcus clade. The list of datasets is given below; the set includes four genomes where translational selection was previously not detected in at least 2 out of 3 of the following studies (dos Reis et al. 2004, Sharp et al. 2005, Carbone et al. 2005): *Pseudomonas aeruginosa*, *Streptomyces coelicolor*, *Mycobacterium tuberculosis* and *Nitrosomonas europaea*.

List of mRNA expression datasets; the NCBI GEO Series ID is given (“GSExxxx”), together with the GEO Samples (“GSMxxxx”) from the series that were averaged to obtain final expression level. These sets contain absolute mRNA signal intensities, from single-channel microarrays (commonly Affymetrix), except *Streptomyces*, *Desulfovibrio* and *Salmonella*, which are from dual-channel microarrays normalized to genomic DNA.

- *Pseudomonas syringae tomato* DC3000: [GSE4848](http://eutils.wip.ncbi.nlm.nih.gov/projects/geo/query/acc.cgi?acc=GSE4848) (GSM109003, 109007, 109009, 109011, 109012, 109014)
- *Mycobacterium tuberculosis* H37Rv: [GSE7588](http://eutils.wip.ncbi.nlm.nih.gov/projects/geo/query/acc.cgi?acc=GSE7588) (GSM183531, 183633)
- *Nitrosomonas europaea*: [GSE10664](http://eutils.wip.ncbi.nlm.nih.gov/projects/geo/query/acc.cgi?acc=GSE10664) (GSM269650, 269651, 269652)
- *Streptococcus mutans*: [GSE6973](http://eutils.wip.ncbi.nlm.nih.gov/projects/geo/query/acc.cgi?acc=GSE6973) (GSM160647 – 160694)
- *Lactobacillus plantarum*: [GSE11383](http://eutils.wip.ncbi.nlm.nih.gov/projects/geo/query/acc.cgi?acc=GSE11383) (GSM287549, 287553, 287554, 287555, 287556, 287559)
- *Bacillus subtilis*: [GSE11937](http://eutils.wip.ncbi.nlm.nih.gov/projects/geo/query/acc.cgi?acc=GSE11937) (GSM299792, 299793, 299794, 299795)
- *Rhodopseudomonas palustris* CGA009: [GSE6221](http://eutils.wip.ncbi.nlm.nih.gov/projects/geo/query/acc.cgi?acc=GSE6221) (GSM143539, 143540, 143541, 143542, 143543, 143544)
- *Thermus thermophilus HB8*: [GSE10368](http://eutils.wip.ncbi.nlm.nih.gov/projects/geo/query/acc.cgi?acc=GSE10368) (GSM261559, 261569, 261594, 261560, 261570, 261595)
- *Bradyrhizobium japonicum*: [GSE12491](http://eutils.wip.ncbi.nlm.nih.gov/projects/geo/query/acc.cgi?acc=GSE12491) (GSM210242, 210243, 210244, 210245)
- *Desulfovibrio vulgaris Hildenborough*: [GSE4447](http://eutils.wip.ncbi.nlm.nih.gov/projects/geo/query/acc.cgi?acc=GSE4447) (GSM101160, 101161, 101162, 101166, 101167, 101168, 101325, 101326, 101330, 101331, 101332, 101333)
- *Haemophilus influenzae*: [GSE5061](http://eutils.wip.ncbi.nlm.nih.gov/projects/geo/query/acc.cgi?acc=GSE5061) (GSM114031, 114032, 114033)
- *Listeria monocytogenes*: [GSE3247](http://eutils.wip.ncbi.nlm.nih.gov/projects/geo/query/acc.cgi?acc=GSE3247) (GSM73161 – 73185)
- *Rhodobacter sphaeroides 2.4.1*: [GSE12269](http://eutils.wip.ncbi.nlm.nih.gov/projects/geo/query/acc.cgi?acc=GSE12269) (GSM308084 - 308107)
- *Staphylococcus aureus* Mu50: [GSE2728](http://eutils.wip.ncbi.nlm.nih.gov/projects/geo/query/acc.cgi?acc=GSE2728) (GSM52706, 52707, 52755, 52724, 52725, 52727, 52744, 52745, 52746)
- *Bifidobacterium longum*: [GSE5865](http://eutils.wip.ncbi.nlm.nih.gov/projects/geo/query/acc.cgi?acc=GSE5865) (GSM136748, 136749, 136750, 136751, 136752)
- *Streptomyces coelicolor*: [GSE7172](http://eutils.wip.ncbi.nlm.nih.gov/projects/geo/query/acc.cgi?acc=GSE7172) (GSM172822, 172823, 172826, 172830)
- *Escherichia coli K12*: [GSE13982](http://eutils.wip.ncbi.nlm.nih.gov/projects/geo/query/acc.cgi?acc=GSE13982) (GSM351280, 351282, 351295, 351297)
- *Salmonella typhimurium LT2*: [GSE4631](http://eutils.wip.ncbi.nlm.nih.gov/projects/geo/query/acc.cgi?acc=GSE4631) (GSM103442, 103443, 103444, 103445, 103450, 103757, 103758, 103759)
- *Pseudomonas aeruginosa*: [GSE4026](http://eutils.wip.ncbi.nlm.nih.gov/projects/geo/query/acc.cgi?acc=GSE4026) (GSM92178, 92179, 92182, 92183, 92186, 92187)

### Normalization of experimental results

The measurements resulting from different experimental essays on *E. coli* mutants were normalized to enable genes to be clustered based on their results across different assays. Additionally, the normalization is meant to facilitate interpretability, where 0 means 'no effect' in all essays (same as *wild-type*), and 1 signifies a 'strong effect'. Values <0 or >1 are possible under certain conditions. The individual essays are normalized as follows:

- protein carbonylation increase: optical density (OD) for each mutant was divided by the OD for the *wild-type*, and 1 subtracted from the result
- measuring ROS:
  - DHR-123 increase: fluorescence was divided by the fluorescence of the *wild-type*, and 1 subtracted from the result
  - CellROX increase: same as DHR-123 increase
- measuring Fe:
  - cellular Fe increase: absorbance for each mutant was divided by the absorbance for the *wild-type*, and 1 subtracted from the result
  - dipyridyl rescue: % survival of each mutant after H_2_O_2_ shock without dipyridyl was subtracted from % survival of the same mutant after H_2_O_2_ shock with dipyridyl, and the result divided by the % survival of the *wild-type* after H_2_O_2_ shock without dipyridyl
- measuring NADPH:
  - cellular NADPH decrease: fluorescence for each mutant was divided by the fluorescence for the *wild-type*, and this value subtracted from 1
  - NADPH rescue: same as dipyridyl rescue

### Supplementary Methods References

Breiman L (2001) Random forests. Machine Learning 45: 5-32.

Carbone A, Kepes F, Zinovyev A (2005) Codon bias signatures, organization of microorganisms in codon space, and lifestyle. Mol Biol Evol 22: 547-561.

Daubin V, Perriere G (2003) G+C3 structuring along the genome: a common feature in prokaryotes. Mol Biol Evol 20: 471-483.

dos Reis M, Savva R, Wernisch L (2004) Solving the riddle of codon usage preferences: a test for translational selection. Nucleic Acids Res 32: 5036-5044.

Fawcett T (2006) An introduction to ROC analysis. Pattern Recognition Letters 27: 861-874.

Hershberg R, Petrov DA (2009) General rules for optimal codon choice. PLoS Genet 5: e1000556.

Karlin S, Mrazek J (2000) Predicted highly expressed genes of diverse prokaryotic genomes. J Bacteriol 182: 5238-5250.

McCutcheon JP, McDonald BR, Moran NA (2009) Origin of an Alternative Genetic Code in the Extremely Small and GC–Rich Genome of a Bacterial Symbiont. PLoS Genetics 5(7):e1000565.

McDonald JH (2008) Sign test. Handbook of Biological Statistics. Baltimore: Sparky House Publishing. pp. 185-189.

Molina N, van Nimwegen E (2008) Universal patterns of purifying selection at noncoding positions in bacteria. Genome Res 18: 148-160.

Nadeau C, Bengio Y (2003) Inference for the generalization error. Machine Learning 52: 239-281.

Rocha EP (2004) The replication-related organization of bacterial genomes. Microbiology 150: 1609-1627.

Sharp PM, Bailes E, Grocock RJ, Peden JF, Sockett RE (2005) Variation in the strength of selected codon usage bias among bacteria. Nucleic Acids Res 33: 1141-1153.

Supek F (2011) The “Fast Random Forest” software. http://fast-random-forest.googlecode.com/

Supek F, Skunca N, Repar J, Vlahovicek K, Smuc T (2010) Translational selection is ubiquitous in prokaryotes. PLoS Genet 6(6): e1001004.

Witten IH, Frank E (2005) Data Mining: Practical machine learning tools and techniques. San Francisco: Morgan Kaufmann.
