# Supplementary material for: Inferring gene function from evolutionary change in signatures of translation efficiency
Source: Genome Biol. 2014 Mar 3;15(3):R44. doi: 10.1186/gb-2014-15-3-r44 (PMC4054840; doi:10.1186/gb-2014-15-3-r44)
Supplement: Additional file 2 — Agreement with expression data for the predictions about highly expressed (HE) genes, and a comparison with the original ‘optimized codon usage’ (OCU) method [[9]].P values are from a Mann-Whitney test for a difference in microarray signal levels between the HE and non-HE genes, or the OCU and non-OCU genes. The ‘ratios’ were calculated between the average microarray signal of the two groups. The ratio of ribosomal proteins versus whole genome is given for a sense of scale; the ribosomal protein genes are expected to be very highly expressed. [file gb-2014-15-3-r44-S2.docx]

**Additional file 2. Agreement to expression data for the predictions about 'highly expressed' (HE) genes, and a comparison to the original ‘OCU’ method.** (‘OCU’ stands for optimized codon usage; from Supek et al., PLOS Genetics 2010). *P* values are from a Mann-Whitney test for a difference of microarray signal levels between the HE and non-HE genes, or the OCU and non-OCU genes. The "ratios" are calculated between the average microarray signal of the two groups. The ratio of ribosomal proteins *vs.* whole genome is given for a sense of scale; the ribosomal protein genes are expected to be very highly expressed.

| organism | # genes* | % HE | P value | HE/non-HE ratio | % OCU | P value | OCU/non-OCU ratio | ribo.prot/all genes ratio |
| --- | --- | --- | --- | --- | --- | --- | --- | --- |
| *Pseudomonas syringae tomato DC3000* | 5280 | 6.5% | 1.2E-13 | 1.87 x | 8.4% | 2.2E-01 | 1.33 x | 4.55 x |
| *Mycobacterium tuberculosis* H37Rv | 3860 | 11.8% | 7.9E-16 | 1.81 x | 17.4% | 9.8E-03 | 1.38 x | 4.31 x |
| *Nitrosomonas europaea* | 2406 | 12.8% | 3.4E-33 | 2.93 x | 15.2% | 8.9E-07 | 1.41 x | 6.77 x |
| *Streptococcus mutans* | 1776 | 8.8% | 5.1E-36 | 1.75 x | 8.1% | 1.9E-11 | 1.42 x | 1.94 x |
| *Lactobacillus plantarum* | 2892 | 6.3% | 1.5E-21 | 2.74 x | 9.8% | 1.9E-05 | 1.68 x | 3.22 x |
| *Bacillus subtilis* | 3809 | 5.4% | 5.1E-27 | 4.75 x | 8.5% | 5.4E-03 | 1.76 x | 9.95 x |
| *Rhodopseudomonas palustris* CGA009 | 4658 | 10.0% | 2.6E-57 | 2.62 x | 12.4% | 4.1E-24 | 2.03 x | 3.56 x |
| *Thermus thermophilus* HB8 | 2122 | 14.8% | 1.1E-18 | 2.72 x | 13.1% | 2.5E-04 | 2.06 x | 6.69 x |
| *Bradyrhizobium japonicum* | 7972 | 8.4% | 6.1E-88 | 2.33 x | 9.1% | 2.8E-33 | 2.14 x | 2.58 x |
| *Desulfovibrio vulgaris Hildenborough* | 3086 | 11.5% | 7.8E-48 | 2.67 x | 13.5% | 4.4E-21 | 2.31 x | 4.72 x |
| *Haemophilus influenzae* | 1574 | 9.3% | 1.4E-53 | 3.78 x | 10.0% | 1.0E-18 | 2.37 x | 4.48 x |
| *Listeria monocytogenes* | 2734 | 9.1% | 5.3E-35 | 4.32 x | 11.9% | 2.7E-05 | 2.74 x | 4.87 x |
| *Rhodobacter sphaeroides* 2-4-1 | 4076 | 8.7% | 1.4E-48 | 5.74 x | 11.8% | 1.0E-06 | 2.84 x | 12.09 x |
| *Staphylococcus aureus* Mu50 | 2498 | 8.0% | 7.4E-35 | 6.76 x | 10.0% | 1.5E-05 | 2.87 x | 2.31 x |
| *Bifidobacterium longum* | 1696 | 10.4% | 5.0E-52 | 6.37 x | 13.3% | 4.2E-16 | 3.11 x | 6.50 x |
| *Streptomyces coelicolor* | 7772 | 6.1% | 1.8E-55 | 4.82 x | 6.9% | 1.6E-38 | 3.13 x | 15.17 x |
| *Escherichia coli* K12 | 3914 | 6.9% | 1.6E-79 | 5.33 x | 7.5% | 4.4E-31 | 3.18 x | 6.81 x |
| *Salmonella typhimurium* LT2 | 4305 | 5.3% | 3.0E-63 | 5.98 x | 7.0% | 1.6E-20 | 3.33 x | 7.71 x |
| *Pseudomonas aeruginosa* | 5426 | 6.8% | 1.4E-60 | 4.76 x | 6.2% | 4.1E-30 | 3.73 x | 7.43 x |
|  |  | *average* | *median* | *average* | *average* | *median* | *Average* | *Average* |
|  |  | 8.8% | 7.8E-48 | 3.90 x | 10.5% | 1.9E-11 | 2.36 x | 6.09 x |

* number of genes at least 80 codons long
