# Supplementary material for: Inferring gene function from evolutionary change in signatures of translation efficiency
Source: Genome Biol. 2014 Mar 3;15(3):R44. doi: 10.1186/gb-2014-15-3-r44 (PMC4054840; doi:10.1186/gb-2014-15-3-r44)
Supplement: Additional file 3 — The relative proportion of highly expressed genes is lower in larger genomes. This correlation was previously explained [9] by different proportions of various gene functional categories in smaller or larger genomes. Many of the functional categories, in turn, tend to have a general preference for higher or lower expression. For instance, larger genomes have a disproportionally increased number of gene regulators, which have a strong tendency to low expression. Smaller genomes, on the other hand, have a higher proportion of ribosomal proteins, whose absolute number is roughly fixed across genomes, regardless of their size. [file gb-2014-15-3-r44-S3.docx]

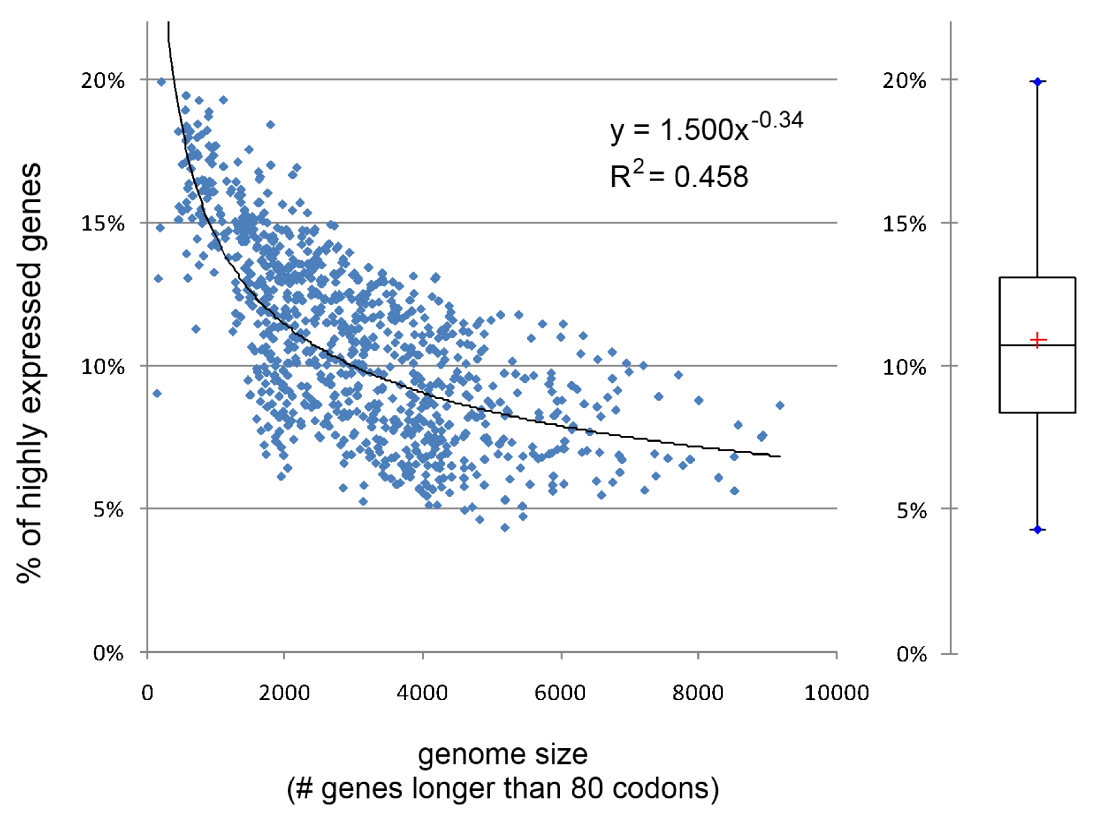


**Additional file 3. The relative proportion of highly expressed genes is lower in larger genomes.** This correlation was previously explained (Supek *et al*. PLOS Genetics 2010) by different proportions of various gene functional categories in smaller or larger genomes. Many of the functional categories, in turn, tend to have a general preference for higher or lower expression. For instance, larger genomes have a disproportionally increased number of gene regulators, which have a strong tendency of being lowly expressed. Smaller genomes, on the other hand, have a higher proportion of ribosomal proteins, whose absolute number is roughly fixed across genomes regardless of their size.
