# Supplementary material for: Inferring gene function from evolutionary change in signatures of translation efficiency
Source: Genome Biol. 2014 Mar 3;15(3):R44. doi: 10.1186/gb-2014-15-3-r44 (PMC4054840; doi:10.1186/gb-2014-15-3-r44)
Supplement: Additional file 4 — Correlations of mRNA 5′ end folding free energies and various codon indices with gene expression levels. The free energies are a measure of the stability of the structures (more negative = more stable) and are calculated in windows of 42 nucleotides in length on the mRNA sequence using the hybrid-ss-min program from UNAFold 3.6 with default parameters, as in [25]. The three 42-nt window positions investigated are: (-4 to 37), found to have a strongest correlation to protein levels [25]; (-20 to 21), a window centered over the start codon; and (-30 to 11), a window centered on the common location of the Shine-Dalgarno sequence at -9 [69]. The -10 kcal/mol figure is the approximate limit for the mRNA folding free energy in 42-nt windows; at negative values below this, the mRNA folding starts to have a considerable effect on translation efficiency [7]. The mRNA coordinates are given relative to the start codon, where 1 is the A in AUG. The codon indices are: CAI [4], B [70], and MILC [5]. RF, probability score obtained from a random forest classifier [9]. All codon indices use the same ‘reference set’ of known highly expressed genes as used in our analyses (see Supplementary Methods in Additional file 1). [file gb-2014-15-3-r44-S4.docx]

**Additional file 4.** **Correlations of mRNA 5' end folding free energies and various codon indices to gene expression levels.** The free energies are a measure of stability of the structures (more negative = more stable) and are calculated in 42-nucleotide windows on the mRNA sequence using the *hybrid-ss-min* program from UnaFold 3.6 with default parameters, as in Kudla *et al*. (*Science* 2009). The three 42-nt window positions investigated are: [-4,37], found to have a strongest correlation to protein levels in Kudla *et al*.; [-20,21], a window centered over the start codon; and [-30,11], a window centered on the common location of the Shine-Dalgarno sequence at -9 (Shultzaberger *et al*. J Mol Biol 2001). The -10 kcal/mol is the approximate limit for the mRNA folding free energy in 42-nt windows below which more negative values the mRNA folding starts to have a considerable effect on translation efficiency (Supek and Šmuc, *Genetics*, 2010). The mRNA coordinates are given relative to the start codon, where 1 is the A in AUG. The codon indices are: CAI (Sharp and Li, *Nucl Acids Res* 1987), B (Karlin and Mrazek, *J Bact* 2000), and MILC (Supek and Vlahoviček, BMC Bioinformatics 2005). RF is the probability score obtained from a Random Forest classifier (Supek *et al.*, PLOS Genetics, 2010). All codon indices use the same 'reference set' of known highly expressed genes as used in our analyses (Supplementary Methods in Additional file 1).

|  |  | Pearson correlation with mRNA levels (from microarray measurements) | | | | | | |  | | |
| --- | --- | --- | --- | --- | --- | --- | --- | --- | --- | --- | --- |
|  |  | codon indices for genes | | | | mRNA folding free energies  in 42 nucleotide windows | | | % genes in genome with mRNA folding free energy  < -10 kcal/mol | | |
| organism | #genes | CAI | B | MILC | RF | [-4,37] | [-20,21] | [-30,11] | [-4,37] | [-20,21] | [-30,11] |
| *Pseudomonas syringae tomato* DC3000 | 5266 | 0.21 | 0.17 | 0.27 | 0.33 | 0.03 | 0.03 | 0.05 | 14% | 13% | 17% |
| *Mycobacterium tuberculosis* H37Rv | 3444 | 0.05 | 0.13 | 0.12 | 0.21 | 0.10 | 0.13 | 0.13 | 48% | 44% | 46% |
| *Nitrosomonas europaea* | 2347 | 0.28 | 0.33 | 0.37 | 0.43 | 0.04 | 0.17 | 0.13 | 9% | 7% | 8% |
| *Streptococcus mutans* | 1741 | 0.45 | 0.29 | 0.49 | 0.34 | -0.08 | -0.02 | 0.04 | 2% | 1% | 1% |
| *Lactobacillus plantarum* | 2646 | 0.22 | 0.20 | 0.22 | 0.32 | -0.07 | -0.03 | -0.01 | 4% | 2% | 3% |
| *Bacillus subtilis* | 3635 | 0.33 | 0.29 | 0.38 | 0.45 | -0.02 | 0.00 | -0.02 | 4% | 3% | 3% |
| *Rhodopseudomonas palustris* CGA009 | 4632 | 0.16 | 0.33 | 0.32 | 0.32 | 0.09 | 0.07 | 0.09 | 31% | 27% | 34% |
| *Thermus thermophilus* HB8 | 2080 | 0.20 | 0.34 | 0.39 | 0.45 | 0.17 | 0.14 | 0.11 | 56% | 56% | 62% |
| *Bradyrhizobium japonicum* | 7853 | -0.08 | 0.10 | 0.02 | 0.14 | 0.04 | 0.05 | 0.05 | 32% | 31% | 35% |
| *Desulfovibrio vulgaris* Hildenborough | 3024 | 0.03 | 0.15 | 0.16 | 0.24 | 0.03 | 0.04 | 0.05 | 24% | 22% | 28% |
| *Haemophilus influenzae* | 1573 | 0.62 | 0.61 | 0.71 | 0.65 | -0.11 | -0.04 | 0.01 | 3% | 2% | 2% |
| *Listeria monocytogenes* | 2729 | 0.35 | 0.40 | 0.43 | 0.43 | -0.03 | -0.04 | 0.01 | 1% | 1% | 1% |
| *Rhodobacter sphaeroides* 2 4 1 | 3839 | 0.20 | 0.40 | 0.50 | 0.60 | 0.08 | 0.12 | 0.12 | 34% | 29% | 40% |
| *Staphylococcus aureus* Mu50 | 2101 | 0.43 | 0.46 | 0.54 | 0.56 | -0.06 | -0.06 | -0.02 | 1% | 1% | 1% |
| *Bifidobacterium longum* | 1687 | 0.41 | 0.49 | 0.52 | 0.56 | 0.05 | 0.07 | 0.08 | 22% | 17% | 18% |
| *Streptomyces coelicolor* | 6984 | 0.12 | 0.24 | 0.30 | 0.49 | 0.01 | 0.03 | 0.04 | 48% | 42% | 45% |
| *Escherichia coli* K12 | 3613 | 0.57 | 0.56 | 0.64 | 0.59 | -0.01 | 0.03 | 0.05 | 9% | 4% | 6% |
| *Salmonella typhimurium* LT2 | 3982 | 0.35 | 0.38 | 0.44 | 0.40 | 0.00 | 0.04 | 0.05 | 8% | 5% | 7% |
| *Pseudomonas aeruginosa* | 5404 | 0.20 | 0.38 | 0.50 | 0.47 | 0.09 | 0.08 | 0.08 | 23% | 19% | 25% |
| ***median of 19 genomes*** | ***3444*** | ***0.22*** | ***0.33*** | ***0.39*** | ***0.43*** | ***0.03*** | ***0.04*** | ***0.05*** | ***14%*** | ***13%*** | ***17%*** |
