# Supplementary material for: Inferring gene function from evolutionary change in signatures of translation efficiency
Source: Genome Biol. 2014 Mar 3;15(3):R44. doi: 10.1186/gb-2014-15-3-r44 (PMC4054840; doi:10.1186/gb-2014-15-3-r44)
Supplement: Additional file 5 — The 100 features describing each organism which were used in the search for the phenotypes predictive of the changes in translation efficiency within clusters of orthologous groups (COGs). All features are binary variables, and can be undefined for some organisms. We included 70 features describing the phylogeny (left/middle columns) and the 6 features describing genome size and GC content (right column, top) to ensure that correlations detected with the remaining 24 features (phenotypes, right column) could not be explained by the phylogeny or the genomic size/GC. #pos, number of organisms marked as positive for a specific feature; #neg, number of organisms marked as negative for a specific feature. [file gb-2014-15-3-r44-S5.docx]

**Additional file 5.** **The 100 features describing each organism which were used in the search for the phenotypes predictive of the changes in translation efficiency within COGs.** All features are binary variables, and can be undefined for some organisms. 70 features describing the phylogeny (left/middle columns) and the 6 features describing genome size and GC content (right column, top) are included to ensure that correlations detected with the remaining 24 features (phenotypes, right column) could not be explained by the phylogeny or the genomic size/GC. "#pos" is the number of organisms marked as positive for the feature, and "#neg" as negative.

| ***domain / phylum / class*** | ***#pos*** | ***#neg*** | ***order (taxonomy)*** | | ***#pos*** | ***#neg*** | | ***genomic features*** | | ***#pos*** | ***#neg*** | |  |
| --- | --- | --- | --- | --- | --- | --- | --- | --- | --- | --- | --- | --- | --- |
| domain: Archaea | 85 | 826 | Actinomycetales | | 83 | 801 | | G+C: above median(>47.5%) | | 425 | 387 | |  |
| domain: Bacteria | 826 | 85 | Alteromonadales | | 28 | 856 | | G+C: high(>59.9%) | | 235 | 576 | |  |
| phylum: Actinobacteria | 97 | 813 | Bacillales | | 46 | 838 | | G+C: low(<37.6%) | | 188 | 624 | |  |
| phylum: Bacteroidetes | 34 | 876 | Bacteroidales | | 10 | 874 | | Genome size: above median(>3.2Mb) | | 454 | 443 | |  |
| phylum: Chlamydiae | 8 | 902 | Bifidobacteriales | | 6 | 878 | | Genome size: large(>4.8Mb) | | 226 | 671 | |  |
| phylum: Chlorobi | 10 | 900 | Burkholderiales | | 47 | 837 | | Genome size: small(<2Mb) | | 195 | 702 | |  |
| phylum: Chloroflexi | 15 | 895 | Campylobacterales | | 18 | 866 | |  | |  |  | |  |
| phylum: Crenarchaeota | 23 | 887 | Chlamydiales | | 8 | 876 | | ***phenotypes*** | | ***#pos*** | ***#neg*** | |  |
| phylum: Cyanobacteria | 28 | 882 | Chlorobiales | | 10 | 874 | | Endospores | | 75 | 337 | |  |
| phylum: Deinococcus-Thermus | 10 | 900 | Chroococcales | | 20 | 864 | | Gram Stain=positive | | 216 | 466 | |  |
| phylum: Euryarchaeota | 58 | 852 | Clostridiales | | 37 | 847 | | Growth in groups | | 61 | 228 | |  |
| phylum: Firmicutes | 145 | 765 | Desulfovibrionales | | 8 | 876 | | Habitat=aquatic(pos) *vs.* terrestrial(neg) | | 167 | 71 | |  |
| phylum: Proteobacteria | 383 | 527 | Enterobacteriales | | 46 | 838 | | Habitat=free-living(pos) *vs.* hostAssociated(neg) | | 238 | 222 | |  |
| phylum: Spirochaetes | 17 | 893 | Flavobacteriales | | 14 | 870 | | Habitat=multiple(pos) *vs.* single(neg) | | 190 | 587 | |  |
| phylum: Tenericutes | 28 | 882 | Halobacteriales | | 12 | 872 | | Halophilic | | 40 | 140 | |  |
| phylum: Thermotogae | 11 | 899 | Lactobacillales | | 36 | 848 | | Motility | | 379 | 231 | |  |
| class: Actinobacteria | 97 | 775 | Legionellales | | 3 | 881 | | Oxygen = aerotolerant (pos) *vs.* strictly anaerobic (neg) | | 514 | 214 | |  |
| class: α-proteobacteria | 107 | 765 | Methanococcales | | 9 | 875 | | Oxygen = facultative aerobe (pos) *vs.* strict aerobe (neg) | | 217 | 296 | |  |
| class: Bacilli | 82 | 790 | Mycoplasmatales | | 22 | 862 | | Psychrophilic | | 25 | 760 | |  |
| class: Bacteroidia | 10 | 862 | Neisseriales | | 5 | 879 | | Radioresistance | | 16 | 887 | |  |
| class: β-proteobacteria | 68 | 804 | Pasteurellales | | 10 | 874 | | Shape = coccus (pos) *vs.* rod (neg) | | 105 | 506 | |  |
| class: Chlamydiae | 8 | 864 | Prochlorales | | 1 | 883 | | Thermophilic | | 142 | 643 | |  |
| class: Chlorobia | 10 | 862 | Pseudomonadales | | 18 | 866 | | Pathogenic in plants | | 23 | 304 | |  |
| class: Clostridia | 61 | 811 | Rhizobiales | | 47 | 837 | | Pathogenic in mammals | | 163 | 166 | |  |
| class: Deinococci | 10 | 862 | Rhodobacterales | | 12 | 872 | | Mammalian pathogen = blood | | 35 | 128 | |  |
| class: δ-proteobacteria | 33 | 839 | Rickettsiales | | 25 | 859 | | Mammalian pathogen =enteric | | 31 | 132 | |  |
| class: ε-proteobacteria | 22 | 850 | Spirochaetales | | 17 | 867 | | Mammalian pathogen = heart | | 8 | 155 | |  |
| class: Flavobacteria | 14 | 858 | Sulfolobales | | 5 | 879 | | Mammalian pathogen = nervous system | | 17 | 146 | |  |
| class: γ-proteobacteria | 152 | 720 | Thermoanaero-bacterales | | 20 | 864 | | Mammalian pathogen = oportunistic/nosocomial | | 16 | 147 | |  |
| class: Halobacteria | 12 | 860 | Thermococcales | | 8 | 876 | | Mammalian pathogen = oral cavity | | 8 | 155 | |  |
| class: Methanococci | 9 | 863 | Thermotogales | | 11 | 873 | | Mammalian pathogen = respiratory | | 43 | 120 | |  |
| class: Methanomicrobia | 14 | 858 | Thiotrichales | | 4 | 880 | | Mammalian pathogen = skin/soft tissues | | 24 | 139 | |  |
| class: Mollicutes | 28 | 844 | Vibrionales | | 9 | 875 | |  | |  |  | |  |
| class: Spirochaetes | 17 | 855 | Xanthomonadales | | 8 | 876 | |  | |  |  | |  |
| class: Thermoprotei | 23 | 849 |  |  | | |  | |  |  | |  | |
| class: Thermotogae | 11 | 861 |  |  | | |  | |  |  | |  | |
