# Supplementary material for: Inferring gene function from evolutionary change in signatures of translation efficiency
Source: Genome Biol. 2014 Mar 3;15(3):R44. doi: 10.1186/gb-2014-15-3-r44 (PMC4054840; doi:10.1186/gb-2014-15-3-r44)
Supplement: Additional file 6 — Genomes for which the optimal codons inferred from over-representation in highly expressed (HE) genes overall did not match the expected optimal codons inferred from the genomic tRNA repertoire. The nine twofold degenerate amino acids were examined. An optimal codon (HE column) was defined as over-represented at P < 0.001 in a Fisher’s exact test on codon counts in HE versus the non-HE genes; a non-significant result means no codon is optimal. The codons expected to be optimal from tRNAs tRNA column) are defined in the genomes in which tRNA genes with only one of the two possible anticodons were present (found by tRNAscan-SE), then the codon matching that anticodon by canonical Watson-Crick pairing was considered tRNA-optimal, and the other codon, which uses wobble pairing, was considered tRNA-suboptimal. The table shows 71 (of the 911 total) genomes for which the optimal and the tRNA-optimal codons disagreed in at least 3 of 9of the testable amino acids (# aa column). In 651/911 genomes, there were 0/9 disagreeing amino acids, and 1/9 for a further 135 genomes. Thus, in the 71 genomes, the expression level-related codon bias did not, overall, clearly relate to the tRNA gene repertoire, and may possibly not reflect translational selection, but rather another, unknown factor. We thus excluded the 71 genomes, and re-ran the subsequent analyses to verify if our findings were robust to inclusion of these genomes (see Additional file 7). [file gb-2014-15-3-r44-S6.docx]

**Additional file 6.** **Genomes where the optimal codons inferred from overrepresentation in HE genes overall do not match the expected optimal codons inferred from the genomic tRNA repertoire.** The nine two-fold degenerate amino acids are examined. An optimal codon ("HE" columns) is defined as overrepresented at P<0.001 in a Fisher's exact test on codon counts in HE vs. the non-HE genes; a non-significant result means no codon is optimal. The codons expected to be optimal from tRNAs ("tRNA" columns) are defined in the genomes where tRNA genes with only one of the two possible anticodons are present (found by tRNAscan-SE); then, the codon matching that anticodon by canonical Watson-Crick pairing is considered tRNA-optimal, and the other that uses wobble pairing is considered tRNA-suboptimal. The table shows 71 (of the 911 total) genomes where the optimal and the tRNA-optimal codons disagree in at least 3 of 9 (column "# aa") of the testable amino acids. (In 651/911 genomes there were 0/9 disagreeing amino acids, and 1/9 for further 135 genomes). Thus, in the 71 genomes, the expression level-related codon bias does not, on overall, clearly relate to the tRNA gene repertoire and may possibly not reflect translational selection, but another, unknown factor. We thus excluded the 71 genomes, and re-run the subsequent analyses to verify our findings are robust to inclusion of these genomes (Additional file 7).

|  |  | Phe |  | Tyr |  | Cys |  | His |  | Gln |  | Asn |  | Lys |  | Asp |  | Glu |  |
| --- | --- | --- | --- | --- | --- | --- | --- | --- | --- | --- | --- | --- | --- | --- | --- | --- | --- | --- | --- |
| Genome | # aa | HE | tRNA | HE | tRNA | HE | tRNA | HE | tRNA | HE | tRNA | HE | tRNA | HE | tRNA | HE | tRNA | HE | tRNA |
| *Ehrlichia canis Jake* | 8 | TTT | TTC | TAT | TAC | TGT | TGC | CAT | CAC | CAG | CAA | AAT | AAC | AAG | - | GAT | GAC | GAG | GAA |
| *Ehrlichia chaffeensis Arkansas* | 8 | TTT | TTC | TAT | TAC | TGT | TGC | CAT | CAC | CAG | CAA | AAT | AAC | AAG | - | GAT | GAC | GAG | GAA |
| *Borrelia turicatae 91E135* | 7 | TTT | TTC | TAT | TAC | - | TGC | CAT | CAC | CAG | CAA | AAT | AAC | AAG | - | GAT | GAC | GAG | GAA |
| *Cand. Blochmannia floridanus* | 7 | TTT | TTC | TAT | TAC | - | TGC | CAT | CAC | CAG | CAA | AAT | AAC | AAG | - | GAT | GAC | GAG | GAA |
| *Ehrlichia ruminantium Welgevonden* | 7 | TTT | TTC | TAT | TAC | - | TGC | CAT | CAC | CAG | CAA | AAT | AAC | AAG | - | GAT | GAC | GAG | GAA |
| *Nitrosomonas europaea ATCC 19718* | 7 | TTT | TTC | TAT | TAC | TGT | TGC | CAT | CAC | CAA | CAA | AAT | AAC | - | AAA | GAT | GAC | GAG | GAA |
| *Borrelia burgdorferi B31* | 7 | TTT | TTC | TAT | TAC | TGT | TGC | CAT | CAC | CAG | CAA | AAT | AAC | AAG | - | GAT | GAC | GAG | - |
| *Cand. Blochmannia vafer BVAF* | 6 | - | TTC | TAT | TAC | TGT | - | CAT | CAC | CAG | CAA | AAT | AAC | AAG | - | GAT | GAC | GAG | GAA |
| *Borrelia recurrentis A1* | 6 | TTT | TTC | TAT | TAC | - | TGC | - | CAC | CAG | CAA | AAT | AAC | AAG | - | GAT | GAC | GAG | GAA |
| *Borrelia hermsii DAH* | 6 | TTT | TTC | TAT | TAC | - | TGC | CAT | CAC | - | CAA | AAT | AAC | AAG | - | GAT | GAC | GAG | GAA |
| *Cand. Azobacteroides pseudotrichonymphae CFP2* | 6 | TTT | TTC | TAT | TAC | TGT | TGC | - | CAC | CAG | - | AAT | AAC | AAG | - | GAT | GAC | GAG | GAA |
| *Wolbachia endosymbiont of D. melanogaster* | 6 | TTT | TTC | TAT | TAC | - | TGC | CAT | CAC | - | CAA | AAT | AAC | AAG | AAA | GAT | GAC | - | GAA |
| *Acidilobus saccharovorans 345 15* | 6 | TTT | TTC | TAT | TAC | TGT | TGC | CAT | CAC | CAA | - | AAT | AAC | AAA | - | GAT | GAC | GAA | - |
| *Chloroherpeton thalassium ATCC 35110* | 6 | TTT | TTC | TAT | TAC | TGT | TGC | CAT | CAC | CAG | - | AAT | AAC | AAG | - | GAT | GAC | - | - |
| *Desulfobulbus propionicus DSM 2032* | 6 | TTT | TTC | TAT | TAC | TGT | TGC | CAT | CAC | CAA | - | AAT | AAC | AAA | - | GAT | GAC | GAA | - |
| *Geobacter metallireducens GS 15* | 6 | TTT | TTC | TAT | TAC | TGT | TGC | CAT | CAC | CAA | CAA | AAT | AAC | - | AAA | GAT | GAC | GAA | GAA |
| *Geobacter uraniireducens Rf4* | 6 | TTT | TTC | TAT | TAC | TGT | TGC | CAT | CAC | CAA | - | AAT | AAC | AAA | AAA | GAT | GAC | GAA | GAA |
| *Methylacidiphilum infernorum V4* | 6 | TTT | TTC | TAT | TAC | TGT | TGC | CAT | CAC | - | - | AAT | AAC | - | - | GAT | GAC | - | - |
| *Nitrosomonas eutropha C91* | 6 | TTT | TTC | TAT | TAC | TGT | TGC | CAT | CAC | CAA | CAA | AAT | AAC | - | AAA | GAT | GAC | - | GAA |
| *Syntrophus aciditrophicus SB* | 6 | TTT | TTC | TAT | TAC | TGT | TGC | CAT | CAC | - | - | AAT | AAC | - | - | GAT | GAC | GAA | - |
| *Thermobaculum terrenum ATCC BAA 798* | 6 | TTT | TTC | TAT | TAC | TGT | TGC | CAT | CAC | - | - | AAT | AAC | - | - | GAT | GAC | GAA | - |
| *Xylella fastidiosa M12* | 6 | TTT | TTC | TAT | TAC | TGT | TGC | CAT | CAC | CAG | - | AAT | AAC | AAG | - | GAT | GAC | - | - |
| *Neorickettsia risticii Illinois* | 5 | TTT | TTC | - | TAC | - | TGC | - | CAC | CAG | CAA | - | AAC | AAG | AAA | GAT | GAC | GAG | GAA |
| *Borrelia duttonii Ly* | 5 | TTT | TTC | TAT | TAC | - | TGC | - | CAC | - | CAA | AAT | AAC | AAG | - | GAT | GAC | GAG | GAA |
| *Thermofilum pendens Hrk 5* | 5 | TTT | TTC | TAT | TAC | TGT | - | CAT | CAC | CAA | - | AAT | - | AAA | AAG | GAT | - | GAA | GAG |
| *Orientia tsutsugamushi Ikeda* | 5 | TTT | TTC | TAT | TAC | - | TGC | - | CAC | - | CAA | AAT | AAC | AAG | AAA | GAT | GAC | - | GAA |
| *Desulfomicrobium baculatum DSM 4028* | 5 | - | TTC | TAT | TAC | TGT | TGC | CAT | CAC | - | - | AAT | AAC | - | - | GAT | GAC | GAA | - |
| *Wolbachia endosymbiont of Culex quinquefasciatus Pel* | 5 | - | TTC | TAT | TAC | TGT | TGC | CAT | CAC | - | CAA | AAT | AAC | - | AAA | GAT | GAC | - | GAA |
| *Bartonella tribocorum CIP 105476* | 5 | TTT | TTC | TAT | TAC | - | TGC | CAT | CAC | CAG | - | AAT | AAC | AAG | - | GAT | GAC | - | GAA |
| *Nitrosococcus oceani ATCC 19707* | 5 | TTT | TTC | TAT | TAC | - | TGC | CAT | CAC | - | - | AAT | AAC | AAG | - | GAT | GAC | GAG | - |
| *Neorickettsia sennetsu Miyayama* | 4 | - | TTC | - | TAC | - | TGC | - | CAC | CAG | CAA | - | AAC | AAG | AAA | GAT | GAC | GAG | GAA |
| *Anaplasma phagocytophilum HZ* | 4 | - | TTC | - | TAC | TGT | TGC | CAT | CAC | CAG | CAA | - | AAC | AAG | - | - | GAC | GAG | GAA |
| *Wolbachia wRi* | 4 | - | TTC | TAT | TAC | - | TGC | - | CAC | - | CAA | AAT | AAC | AAG | AAA | GAT | GAC | - | GAA |
| *gamma proteobacterium HdN1* | 4 | TTC | TTC | - | TAC | TGT | TGC | CAT | CAC | - | CAA | - | AAC | AAG | AAA | GAT | GAC | - | GAA |
| *Cand. Phytoplasma mali* | 4 | TTT | TTC | TAT | TAC | - | TGC | - | CAC | - | CAA | - | AAC | AAG | AAA | GAT | GAC | - | GAA |
| *Metallosphaera sedula DSM 5348* | 4 | - | TTC | TAT | TAC | - | - | CAT | CAC | CAA | - | AAT | AAC | - | - | GAT | GAC | GAA | - |
| *Cand. Nitrospira defluvii* | 4 | TTT | TTC | - | TAC | - | TGC | CAT | CAC | - | - | AAT | AAC | AAG | - | GAT | GAC | - | - |
| *Sulfolobus acidocaldarius DSM 639* | 4 | TTT | TTC | TAT | - | - | TGC | CAT | CAC | - | - | AAT | AAC | AAA | - | GAT | GAC | GAA | - |
| *Bacteroides helcogenes P 36 108* | 4 | TTT | TTC | - | TAC | TGT | TGC | - | CAC | CAA | - | AAT | AAC | - | - | GAT | GAC | GAA | - |
| *Nitrosococcus halophilus Nc4* | 4 | TTT | TTC | - | TAC | TGT | TGC | - | CAC | CAG | - | AAT | AAC | AAG | - | GAT | GAC | - | - |
| *Borrelia afzelii PKo* | 4 | TTT | TTC | TAT | TAC | - | TGC | - | CAC | - | CAA | AAT | AAC | AAG | - | GAT | GAC | GAG | - |
| *Borrelia garinii PBi* | 4 | TTT | TTC | TAT | TAC | - | TGC | - | CAC | - | CAA | AAT | AAC | AAG | - | GAT | GAC | GAG | - |
| *Sulfolobus islandicus L D 8 5* | 4 | TTT | TTC | TAT | TAC | - | - | - | CAC | - | - | AAT | AAC | - | - | GAT | GAC | GAA | - |
| *Sulfolobus solfataricus P2* | 4 | TTT | TTC | TAT | TAC | - | - | - | CAC | - | - | AAT | AAC | - | - | GAT | GAC | GAA | - |
| *Anaplasma centrale Israel* | 3 | - | TTC | - | TAC | TGT | TGC | - | CAC | CAG | CAA | - | AAC | AAG | - | - | GAC | GAG | GAA |
| *Anaplasma marginale Florida* | 3 | - | TTC | - | TAC | TGT | TGC | - | CAC | CAG | CAA | - | AAC | AAG | - | - | GAC | GAG | GAA |
| *Bartonella clarridgeiae 73* | 3 | TTT | TTC | - | TAC | - | TGC | - | CAC | CAG | CAA | - | AAC | AAG | - | - | GAC | GAG | GAA |
| *Bartonella henselae Houston 1* | 3 | - | TTC | - | TAC | - | TGC | - | CAC | CAG | - | AAT | AAC | AAG | - | GAT | GAC | GAG | GAA |
| *Bartonella quintana Toulouse* | 3 | - | TTC | - | TAC | - | TGC | - | CAC | CAG | - | AAT | AAC | AAG | - | GAT | GAC | GAG | GAA |
| *Caldivirga maquilingensis IC 167* | 3 | - | TTC | TAT | TAC | - | TGC | - | CAC | CAG | - | AAT | AAC | AAG | - | GAT | GAC | GAG | - |
| *Cand. Liberibacter solanacearum CLso ZC1* | 3 | TTT | TTC | TAT | TAC | - | TGC | - | CAC | CAG | - | - | - | AAG | - | GAT | GAC | - | GAA |
| *Cellulophaga algicola DSM 14237* | 3 | TTC | TTC | TAC | TAC | TGT | TGC | CAC | CAC | - | CAA | AAC | AAC | AAG | AAA | GAT | GAC | - | GAA |
| *Chlorobium phaeobacteroides DSM 266* | 3 | - | TTC | - | TAC | TGT | TGC | - | CAC | CAG | - | AAT | AAC | AAG | - | GAT | GAC | GAG | - |
| *Clostridium kluyveri DSM 555* | 3 | - | TTC | TAT | TAC | - | TGC | - | CAC | - | - | AAT | AAC | AAG | - | GAT | GAC | GAA | - |
| *Desulfotalea psychrophila LSv54* | 3 | TTC | TTC | - | TAC | TGT | TGC | - | CAC | - | CAA | - | AAC | AAG | AAA | GAT | GAC | - | - |
| *Dictyoglomus thermophilum H 6 12* | 3 | TTT | TTC | TAT | TAC | - | TGC | - | CAC | - | - | - | AAC | AAG | - | GAT | GAC | GAG | - |
| *Dictyoglomus turgidum DSM 6724* | 3 | - | TTC | TAT | TAC | - | TGC | CAT | CAC | - | - | - | AAC | - | - | GAT | GAC | - | - |
| *Geobacter sulfurreducens PCA* | 3 | TTC | TTC | TAT | TAC | - | TGC | - | CAC | - | CAA | AAT | AAC | - | AAA | GAT | GAC | GAA | GAA |
| *Helicobacter felis ATCC 49179* | 3 | - | TTC | - | TAC | TGT | TGC | - | CAC | - | CAA | AAT | AAC | - | AAA | GAT | GAC | GAA | GAA |
| *Ignisphaera aggregans DSM 17230* | 3 | TTT | TTC | - | TAC | - | - | - | CAC | - | - | AAT | AAC | - | - | GAT | GAC | - | - |
| *Nitrosococcus watsoni C 113* | 3 | TTT | TTC | - | TAC | - | TGC | - | CAC | - | - | AAT | AAC | AAG | - | GAT | GAC | - | - |
| *Pyrobaculum arsenaticum DSM 13514* | 3 | TTT | TTC | - | TAC | TGT | TGC | - | CAC | - | - | - | AAC | AAG | - | GAT | GAC | - | - |
| *Pyrobaculum islandicum DSM 4184* | 3 | TTT | TTC | TAT | TAC | TGT | - | CAT | CAC | - | - | AAT | - | - | - | GAT | - | GAA | - |
| *Rickettsia peacockii Rustic* | 3 | TTT | TTC | - | TAC | - | TGC | - | CAC | CAA | CAA | AAT | AAC | AAA | AAA | GAT | GAC | GAA | GAA |
| *Rickettsia rickettsii Sheila Smith* | 3 | TTT | TTC | - | TAC | - | TGC | - | CAC | - | CAA | AAT | AAC | - | AAA | GAT | GAC | GAA | GAA |
| *Rickettsia typhi Wilmington* | 3 | TTT | TTC | - | TAC | - | TGC | - | CAC | - | CAA | AAT | AAC | - | AAA | GAT | GAC | - | GAA |
| *Spirochaeta thermophila DSM 6192* | 3 | - | TTC | TAT | TAC | - | TGC | - | CAC | CAG | - | AAT | AAC | - | - | GAT | GAC | - | - |
| *Sulfolobus tokodaii 7* | 3 | - | TTC | TAT | TAC | - | TGC | - | CAC | CAA | - | AAT | AAC | AAA | - | GAT | GAC | GAA | - |
| *Thermoproteus neutrophilus V24Sta* | 3 | TTT | TTC | TAT | TAC | - | - | - | CAC | - | CAG | - | - | AAG | AAG | GAT | GAC | GAG | GAG |
| *Thiomicrospira crunogena XCL 2* | 3 | TTC | TTC | - | TAC | TGT | TGC | - | CAC | - | CAA | AAC | AAC | AAG | AAA | GAT | GAC | - | GAA |
| *Wolbachia endosymbiont TRS of Brugia malayi* | 3 | TTT | TTC | TAT | TAC | - | TGC | - | CAC | - | CAA | AAT | AAC | - | AAA | - | GAC | - | GAA |
