# Supplementary material for: Inferring gene function from evolutionary change in signatures of translation efficiency
Source: Genome Biol. 2014 Mar 3;15(3):R44. doi: 10.1186/gb-2014-15-3-r44 (PMC4054840; doi:10.1186/gb-2014-15-3-r44)
Supplement: Additional file 7 — Robustness of the 200 discovered clusters of orthologous groups (COGs)-phenotype links to the exclusion of 71 genomes for which codon biases were not clearly related to the tRNA gene repertoires. Excluded genomes are listed in Additional file 6. (A) The log2 enrichment of the 200 COG-phenotype links with the full set of 911 genomes, and after exclusion of the 71 genomes. (B) Same as (A), but limited to the links that we experimentally validated. In the original analysis, a threshold of log2 enrichment of ≥1 or ≤-1 was a requirement for calling the 200 COG-phenotype links; after excluding the 71 genomes, 195 COG-phenotype links still met this criterion. (C) The log10P value for significance of the enrichment/depletion (two-tailed Fisher’s exact test), again compared between the original and the reduced genome sets. (D) Same as (C), but only for the COG-phenotype links with log10P≥-6. In the original analysis, log10P≤-2 was required for calling the 200 COG-phenotype links; after excluding the 71 genomes, 173/200 links still had log10P≤-2, and 185/200 still had log10P≤-1.7 (P < 0.02). [file gb-2014-15-3-r44-S7.docx]

**Additional file 7.** **Robustness of the 200 discovered COG-phenotype links to exclusion of 71 genomes where codon biases were not clearly related to the tRNA gene repertoires.** Excluded genomes are listed in Additional file 6. (A) The log_2_ enrichment of the 200 COG-phenotype links with the full set of 911 genomes, and after excluding the 71 genomes. (B) Same as A, but limited to the links that we experimentally validated. In the original analysis, a threshold of log_2_ enrichment ≥ 1 or ≤ -1 was a requirement for calling the 200 COG-phenotype links; after excluding the 71 genomes, 195 COG-phenotype links still meet this criterion. (C) The log_10_ P value for significance of the enrichment/depletion (two-tailed Fisher's exact test), again compared between the original and the reduced genome sets. (D) same as C, but only for the COG-phenotype links with log_10_P ≥ -6. In the original analysis, log_10_P ≤ -2 was required for calling the 200 COG-phenotype links; after excluding the 71 genomes, 173/200 links still have log_10_P ≤ -2, and 185/200 still have log_10_P ≤ -1.7 (P<0.02).
