# Supplementary material for: Inferring gene function from evolutionary change in signatures of translation efficiency
Source: Genome Biol. 2014 Mar 3;15(3):R44. doi: 10.1186/gb-2014-15-3-r44 (PMC4054840; doi:10.1186/gb-2014-15-3-r44)
Supplement: Additional file 8 — Survival of Escherichia coli deletion mutants after oxidative stress induced by different hydrogen peroxide concentrations. Survival after heat and osmotic shock is given for comparison. Deleted genes are on the x axis. The y axis shows the survival of the mutant, normalized to the survival of the wild type (w.t.) under the same conditions, which was 45.6% for 0.5 mM H2O2, 13.8% for 2.5 mM H2O2, 4.2% for 20 mM H2O2, 23.6% for heat shock, and 21.3% for osmotic shock. The lon and recA mutants are shown separately as they exhibited a non-specific stress response, being sensitive also to osmotic and heat stress. sodA is a known oxidative stress defense gene, serving as a positive control. [file gb-2014-15-3-r44-S8.docx]

**Additional file 8. Survival of *E. coli* deletion mutants after oxidative stress induced by different hydrogen peroxide concentrations.** Survival after heat and osmotic shocks is given for comparison. Deleted genes are on the *x* axis. The *y* axis shows the mutants' survival, normalized to the survival of the *w.t.* under the same conditions, which is 45.6% for the 0.5 mM, 13.8% for the 2.5 mM and 4.2% for the 20 mM H_2_O_2_, 23.6% for the heat shock and 21.3% for the osmotic shock. *lon* and *recA* mutants are separated for showing a non-specific response, being also sensitive to oxidative and heat stress. *sodA* is a known oxidative stress defense gene, serving as a positive control.
