# Supplementary material for: Inferring gene function from evolutionary change in signatures of translation efficiency
Source: Genome Biol. 2014 Mar 3;15(3):R44. doi: 10.1186/gb-2014-15-3-r44 (PMC4054840; doi:10.1186/gb-2014-15-3-r44)
Supplement: Additional file 9 — Complementing Escherichia coli deletion mutants with wild-type genes. Survival of E. coli deletion mutants in the putative oxidative stress response genes with and without the corresponding genes expressed from a plasmid. [file gb-2014-15-3-r44-S9.docx]

**Additional file 9. Complementing *E. coli* deletion mutants with wild-type genes.** Survival of *E. coli* deletion mutants in the putative oxidative stress response genes with and without the corresponding genes expressed from a plasmid, at two different H_2_O_2_ concentrations (2.5 mM and 20 mM). All 20 mM measurements are from at least two experiments in duplicate, and for 2.5 mM, from three experiments in duplicate.

| 2.5 mM (default) | **complementation** |  |  | **no complementation** | |
| --- | --- | --- | --- | --- | --- |
| **strain** | **average %survival** | **st. dev.** | **as % of w.t. survival** | **average %survival** | **st. dev.** |
| **wt** | N/A | N/A | N/A | 13.78 | 0.34 |
| **clpA** | 13.38 | 0.64 | 97% | 4.82 | 0.21 |
| **cysD** | 13.87 | 0.44 | 101% | 2.42 | 0.19 |
| **fre** | 13.63 | 0.46 | 99% | 3.36 | 0.11 |
| **gpmM** | 13.52 | 0.45 | 98% | 5.14 | 0.12 |
| **icd** | 13.62 | 0.44 | 99% | 2.43 | 0.28 |
| **lon** | 13.28 | 0.40 | 96% | 0.50 | 0.09 |
| **lpd** | 13.20 | 0.54 | 96% | 3.18 | 0.32 |
| **napF** | 13.15 | 0.51 | 95% | 0.83 | 0.09 |
| **recA** | 13.18 | 0.50 | 96% | 4.27 | 0.16 |
| **rseC** | 13.55 | 0.35 | 98% | 1.50 | 0.10 |
| **soda** | 13.62 | 0.50 | 99% | 2.38 | 0.17 |
| **sufD** | 12.98 | 0.72 | 94% | 2.48 | 0.12 |
| **yaaU** | 13.23 | 0.64 | 96% | 1.38 | 0.10 |
| **ybeQ** | 13.38 | 0.33 | 97% | 0.80 | 0.09 |
| **ybhJ** | 13.47 | 0.49 | 98% | 3.44 | 0.16 |
| **yidH** | 13.57 | 0.51 | 98% | 2.34 | 0.13 |

| 20 mM (high) | **complementation** |  |  | **no complementation** | |
| --- | --- | --- | --- | --- | --- |
| **strain** | **average %survival** | **st. dev.** | **as % of w.t. survival** | **average %survival** | **st. dev.** |
| **wt** | N/A | N/A | N/A | 4.16 | 0.17 |
| **clpA** | 4.00 | 0.26 | 96% | 0.74 | 0.11 |
| **cysD** | 3.98 | 0.15 | 96% | 0.00 | 0.00 |
| **fre** | 4.10 | 0.29 | 99% | 0.00 | 0.00 |
| **gpmM** | 3.98 | 0.25 | 96% | 0.00 | 0.00 |
| **icd** | 4.03 | 0.24 | 97% | 0.00 | 0.00 |
| **lon** | 3.81 | 0.09 | 92% | 0.16 | 0.12 |
| **lpd** | 4.10 | 0.18 | 99% | 1.11 | 0.18 |
| **napF** | 4.05 | 0.35 | 97% | 0.38 | 0.07 |
| **recA** | 3.93 | 0.26 | 94% | 2.23 | 0.46 |
| **rseC** | 4.11 | 0.18 | 99% | 0.80 | 0.09 |
| **sodA** | 3.93 | 0.17 | 94% | 0.66 | 0.10 |
| **sufD** | 4.10 | 0.29 | 99% | 0.00 | 0.00 |
| **yaaU** | 4.03 | 0.17 | 97% | 0.87 | 0.17 |
| **ybeQ** | 3.93 | 0.17 | 94% | 0.36 | 0.12 |
| **ybhJ** | 3.88 | 0.10 | 93% | 0.15 | 0.14 |
| **yidH** | 3.93 | 0.21 | 94% | 0.00 | 0.00 |
