# Supplementary material for: Inferring gene function from evolutionary change in signatures of translation efficiency
Source: Genome Biol. 2014 Mar 3;15(3):R44. doi: 10.1186/gb-2014-15-3-r44 (PMC4054840; doi:10.1186/gb-2014-15-3-r44)
Supplement: Additional file 10 — Supporting evidence for putative oxidative stress genes. A survey of the evidence in the literature offering support for the involvement of sufD, clpA, icd, gpmM, lpd, and cysD genes in oxidative stress resistance of various organisms. [file gb-2014-15-3-r44-S10.docx]

**Additional file 10.** **Supporting evidence for putative oxidative stress genes.** A survey of the evidence in the literature offering support for the involvement of *sufD*, *clpA*, *icd*, *gpmM*, *lpd* and *cysD* genes in oxidative stress resistance of various organisms.

| gene | organism | Reference |
| --- | --- | --- |
| direct evidence in *E. coli* or homologs in other bacteria | | |
| *sufD* | *E. coli* | [Tokumoto *et al.*, J Biochem 2004](http://www.ncbi.nlm.nih.gov/pubmed/15496591)  [Saini *et al.*, Biochemistry 2010](http://www.ncbi.nlm.nih.gov/pubmed/20857974) |
|  | *Erwinia chrysanthemi* | [Nachin *et al.*, Mol Microbiol 2001](http://www.ncbi.nlm.nih.gov/pubmed/11251816) |
| *gpmM* | *Mycobacterium tuberculosis* | [Chaturvedi *et al.*, JBC 2010](http://www.jbc.org/content/285/40/30389.full) |
| *clpA* | *Helicobacter pylori* | [Loughlin *et al.*, Microb Pathog 2009](http://www.ncbi.nlm.nih.gov/pubmed/18992803) |
|  | *Brucella suis* | [Ekaza *et al.,* J Bact 2001](http://www.ncbi.nlm.nih.gov/pubmed/11274130?dopt=Abstract) |
| direct evidence for homologs in eukaryotes | | |
| *icd* | mouse | [Lee *et al.*, Free Radical Biol Med 2002](http://www.sciencedirect.com/science/article/pii/S0891584902008158) |
| *gpmM* | mouse | [Kondoh *et al.*, Cancer Res 2005](http://cancerres.aacrjournals.org/content/65/1/177.long) |
| evidence of regulation under aerobiosis or oxidative stress | | |
| *lpd* | E. coli | [Cunningham *et al*., FEMS Microbiol Lett 1998](http://www.sciencedirect.com/science/article/pii/S0378109798005060) |
| *cysD* | *Mycobacterium tuberculosis* | [Pinto *et al*., Microbiology 2004](http://mic.sgmjournals.org/content/150/6/1681.long) |
|  | S*hewanella oneidensis* | [Brown *et al*., Mol Cell Proteomics 2006](http://www.mcponline.org/content/5/6/1054.long#T3) |
