# Supplementary material for: Inferring gene function from evolutionary change in signatures of translation efficiency
Source: Genome Biol. 2014 Mar 3;15(3):R44. doi: 10.1186/gb-2014-15-3-r44 (PMC4054840; doi:10.1186/gb-2014-15-3-r44)
Supplement: Additional file 11 — Functional interactions with known oxidative stress genes. Predicted functional interactions between 34 clusters of orthologous groups (COGs) we found to have codon adaptation that correlates with the aerobic lifestyle, and 30 COGs encoding known Escherichia coli oxidative stress response proteins. The predicted interactions are from the STRING v9.0 database, using exclusively co-expression (top part of table), or exclusively text mining (bottom part) evidence. Only interactions marked as high confidence by STRING (confidence ≥0.7) are shown. [file gb-2014-15-3-r44-S11.docx]

**Additional file 11.** **Functional interactions with known oxidative stress genes.** Predicted functional interactions between 34 COGs we found to have codon adaptation which correlates to the aerobic lifestyle, and 30 COGs encoding known *E. coli* oxidative stress response proteins. The predicted interactions are from the STRING v9.0 database, using exclusively co-expression (top part of table), or exclusively text mining (bottom part) evidence. Only interactions marked as high-confidence by STRING (confidence ≥ 0.7) are shown.

| **Interactor 1: COG differentially expressed in aerobes *vs*. anaerobes** | ***E. coli* gene(s)** | **Interactor 2: known *E. coli* oxidative stress response** | ***E. coli* gene(s)** | **interaction**  **confidence** |
| --- | --- | --- | --- | --- |
| INFERRED FROM CO-EXPRESSION | | | | |
| COG:0543 2-polyprenylphenol hydroxylase and related flavodoxin oxidoreductases | *fre* | COG:0753 Catalase | *katE* | 0.952 |
| COG:0126 3-phosphoglycerate kinase | *pgk* | COG:1225 Peroxiredoxin | *bcp* | 0.908 |
| COG:0543 2-polyprenylphenol hydroxylase and related flavodoxin oxidoreductases | *fre* | COG:450 Peroxiredoxin | *ahpC* | 0.832 |
| COG:0539 Ribosomal protein S1 | *rpsA* | COG:1225 Peroxiredoxin | *bcp* | 0.818 |
| COG:0543 2-polyprenylphenol hydroxylase and related flavodoxin oxidoreductases | *fre* | COG:0695 Glutaredoxin and related proteins | *grxA, grxC* | 0.814 |
| COG:0126 3-phosphoglycerate kinase | *pgk* | COG:0450 Peroxiredoxin | *ahpC* | 0.785 |
| COG:0126 3-phosphoglycerate kinase | *pgk* | COG:0376 Catalase (peroxidase I) | *katG, katP* | 0.736 |
| INFERRED FROM TEXT MINING | | | | |
| COG:1048 Aconitase A | *ybhJ, acnA* | COG:0605 Superoxide dismutase | *sodA, sodB* | 0.975 |
| COG:1048 Aconitase A | *ybhJ, acnA* | COG:0753 Catalase | *katE* | 0.953 |
| COG:2235 Arginine deiminase | (no genes) | COG:0605 Superoxide dismutase | *sodA, sodB* | 0.949 |
| COG:1048 Aconitase A | *ybhJ, acnA* | COG:0276 Protoheme ferro-lyase (ferrochelatase) | *hemH* | 0.890 |
| COG:1048 Aconitase A | *ybhJ, acnA* | COG:0386 Glutathione peroxidase | *btuE* | 0.880 |
| COG:1048 Aconitase A | *ybhJ, acnA* | COG:0735 Fe2+/Zn2+ uptake regulation proteins | *fur* | 0.875 |
| COG:1048 Aconitase A | *ybhJ, acnA* | COG:2032 Cu/Zn superoxide dismutase | *sodC* | 0.842 |
| 0538 Isocitrate dehydrogenases | *icd* | COG:0753 Catalase | *katE* | 0.778 |
| 0175 3'-phosphoadenosine 5'-phosphosulfate sulfotransferase (PAPS reductase)/FAD synthetase and related enzymes | *cysD, cysH* | COG:0492 Thioredoxin reductase | *trxB* | 0.779 |
| 0538 Isocitrate dehydrogenases | *icd* | COG:0605 Superoxide dismutase | *sodA, sodB* | 0.810 |
