# Supplementary material for: Inferring gene function from evolutionary change in signatures of translation efficiency
Source: Genome Biol. 2014 Mar 3;15(3):R44. doi: 10.1186/gb-2014-15-3-r44 (PMC4054840; doi:10.1186/gb-2014-15-3-r44)
Supplement: Additional file 12 — Literature data suggesting putative antioxidant mechanism of action assignments. Listed for the sufD, fre, rseC, gpmM, lpd, and icd genes. [file gb-2014-15-3-r44-S12.docx]

**Additional file 12.** **Literature data suggesting putative antioxidant mechanism-of-action assignments.** Listed for the *sufD*, *fre*, *rseC*, *gpmM*, *lpd* and *icd* genes.

| gene | Description | reference |
| --- | --- | --- |
| *sufD* | part of the sufBCD system for assembly of Fe-S clusters under oxidative stress | [Jang and Imlay, Mol Microbiol 2010](http://www.ncbi.nlm.nih.gov/pubmed/21143317) |
|  | required for in vivo iron acquisition, but not during Fe-S cluster assembly or cluster maturation | [Saini *et al*, Biochemistry 2010](http://www.ncbi.nlm.nih.gov/pubmed/20857974) |
| *fre* | transfers electrons to reduce an Fe(III) center of ribonucleotide reductase, thereby activating the enzyme | [Fontecave *et al*, JBC 1987](http://www.jbc.org/content/262/25/12325.full.pdf+html) [Coves *et al*, JBC 1993](http://www.ncbi.nlm.nih.gov/pubmed/8360156) |
|  | reduces and mobilizes iron from ferrisiderophores | [Coves & Fontecave, Eur J Biochem 1993](http://onlinelibrary.wiley.com/doi/10.1111/j.1432-1033.1993.tb17591.x/full) |
| *rseC* | reduces the 2Fe-2S cluster in the redox-sensitive transcriptional activator SoxR; oxidised form of SoxR may be unstable | [Koo *et al*, EMBO J 2003](http://www.ncbi.nlm.nih.gov/pubmed/12773378) |
| *gpmM* | a glycolysis protein; overexpressing it causes an increased glycolytic flux | [Kondoh *et al*, 2005 Cancer Res](http://cancerres.aacrjournals.org/content/65/1/177.long)  [Kondoh, 2008 Experimental Cell Res](http://www.sciencedirect.com/science/article/pii/S0014482708001353) |
| *lpd* | encodes a subunit of the NADH-producing enzymes pyruvate dehydrogenase and 2-oxoglutarate dehydrogenase | [Kim *et al*, J Bact 2008](http://jb.asm.org/content/190/11/3851.full?view=long&pmid=18375566) [Bunik & Fernie, Biochem J 2009](http://www.biochemj.org/bj/422/0405/bj4220405.htm) |
|  | under stress conditions, conversion of NADH into the protective NADPH via NAD-kinase is enhanced | [Grose *et al*, PNAS 2006](http://www.ncbi.nlm.nih.gov/pubmed/16682646) |
| *icd* | an NADPH-generating enzyme of the citric acid cycle; also produces alpha-ketoglutarate which can serve as an antioxidant | [Mailloux *et al*, PLoS One 2007](http://www.plosone.org/article/info%253Adoi%252F10.1371%252Fjournal.pone.0000690) |
