# Supplementary material for: Inferring gene function from evolutionary change in signatures of translation efficiency
Source: Genome Biol. 2014 Mar 3;15(3):R44. doi: 10.1186/gb-2014-15-3-r44 (PMC4054840; doi:10.1186/gb-2014-15-3-r44)
Supplement: Additional file 13 — The functional context of the 13 Escherichia coli gene representatives of the clusters of orthologous groups (COGs) differentially expressed in aerobic microbes. The genes recA and lon are not shown because their deletion mutants showed non-specific stress sensitivity (Figure 3). Lines represent the predicted functional interactions from the STRING 9.0 database (medium confidence level, ≥0.4), while dots represent all proteins interacting with at least 1 of the 13 proteins. A large, highly interconnected set of interacting ribosomal proteins is not shown for clarity. The larger, colored dots are proteins annotated with one of the selected functional categories in E. coli (right panel). Hollow circles in fre or rseC or thick border in napF denote putative assignments we inferred for these genes from the literature; all other functional annotations were from the Uniprot-GOA (Gene Ontology Annotation) database. All shown functional categories were found to be enriched among the 13 proteins plus interactors at P < 0.05 (hypergeometric distribution, corrected for multiple testing) using GeneCodis 2.0. Proximity of the circles in the figure roughly corresponds to their functional similarity, as optimized by the Edge Weighted Spring Embedded layout in Cytoscape 2.8.1, edge weights being derived from interaction confidence levels in STRING. [file gb-2014-15-3-r44-S13.docx]

**
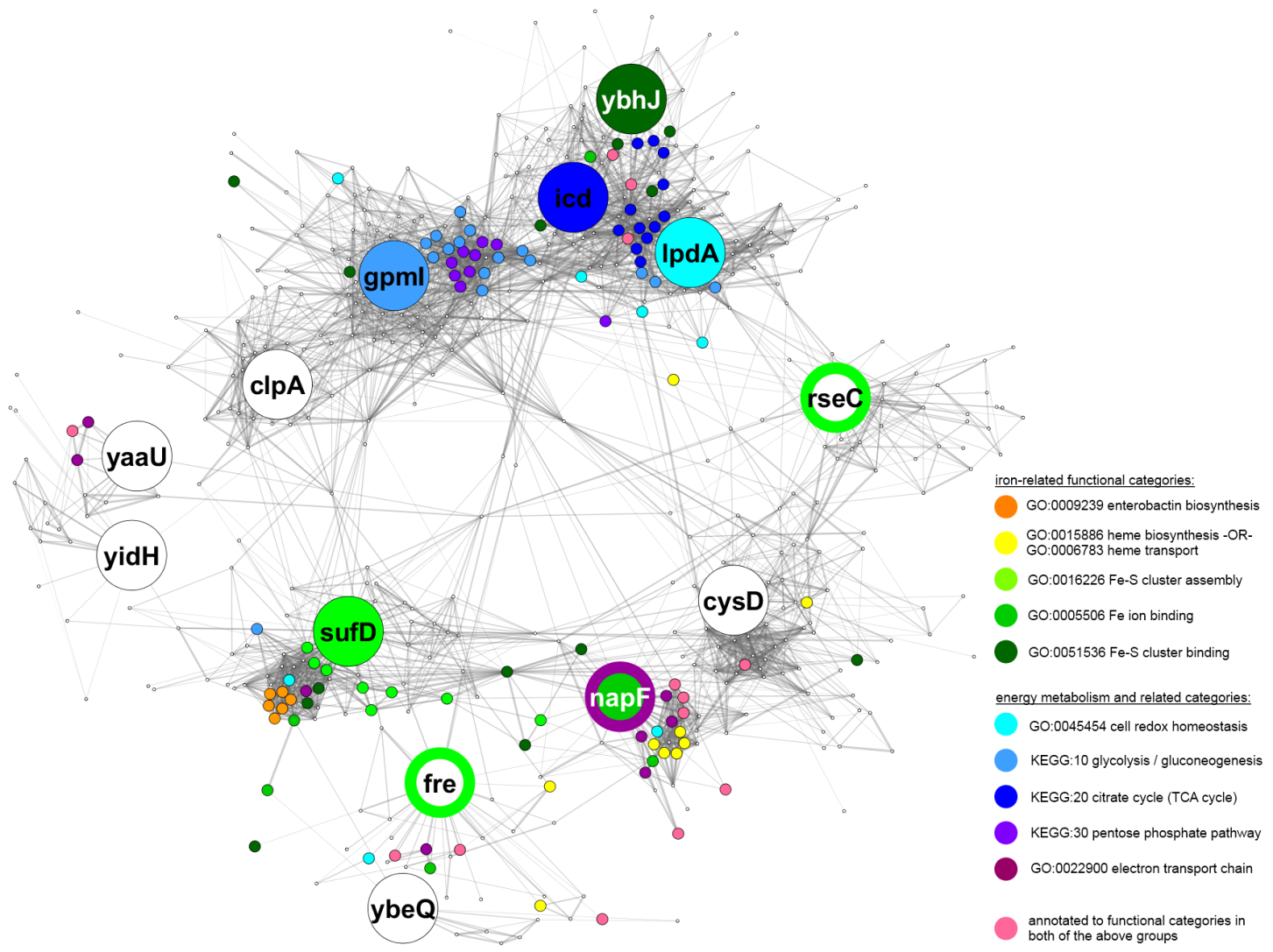
**

**Additional file 13. The functional context of the 13 *E. coli* gene representatives of the COGs differentially expressed in aerobic microbes.** The genes *recA* and *lon* are not shown as their deletion mutants show nonspecific stress sensitivity (Figure 3). Lines represent the predicted functional interactions from the STRING 9.0 database (medium confidence level, ≥0.4), while dots represent all proteins interacting with at least one of the 13 proteins. A large, highly interconnected set of interacting ribosomal proteins is not shown for clarity. The larger, colored dots are proteins annotated with one of the selected functional categories in *E. coli* (right panel). Hollow circles in *fre* or *rseC* or thick border in *napF* denote putative assignments we inferred for the genes from the literature; all other functional annotations were from the EBI’s Uniprot-GOA database. All shown functional categories were found to be enriched among the 13 proteins+interactors at *P*<0.05 (hypergeometric distribution, corrected for multiple testing) using GeneCodis 2.0. Proximity of the discs in the figure roughly corresponds to their functional similarity, as optimized by the “Edge Weighted Spring Embedded” layout in Cytoscape 2.8.1, edge weights being derived from interaction confidence levels in STRING.
