# Supplementary material for: Inferring gene function from evolutionary change in signatures of translation efficiency
Source: Genome Biol. 2014 Mar 3;15(3):R44. doi: 10.1186/gb-2014-15-3-r44 (PMC4054840; doi:10.1186/gb-2014-15-3-r44)
Supplement: Additional file 14 — Profiles of folding free energies in 42-nucleotide windows along the clpS and yjjB gene mRNAs. The x axes show the starting coordinate (in nucleotides) of the 42-nt window. The folding free energies were calculated using the hybrid-ss-min program from UNAFold 3.6 software with default parameters. Alongside each Escherichia coli gene (marked ‘w.t.’), three variants are given with introduced synonymous changes that reduce codon optimality (Figure 5); the number given after the word ‘variant’ is the number of codons that have been altered with respect to the wild type. A 14-nt ribosome binding site sequence, AGGAGGUAAAACAU, was prepended before the AUG start codon when determining the folding free energies, as was the case for the actual genes. For each variant, Pearson’s correlation coefficient, r, and the root mean square deviation (RMSD) are given as measures of similarities of their folding free energy profiles to the wild-type sequence. [file gb-2014-15-3-r44-S14.docx]

**Additional file 14.** **Profiles of folding free energies in 42-nt windows along the *clpS* and *yjjB* gene mRNAs.** The *x* axes show the starting coordinate (in nucleotides) of the 42-nt window. The folding free energies were calculated using the *hybrid-ss-min* program from UNAFold 3.6 software with default parameters. Alongside each *E. coli* gene (marked "w.t."), three variants are given with introduced synonymous changes that reduced codon optimality (Fig. 5); the number given after the word "variant" is the number of codons that have been altered, with respect to the wild type. A 14-nt ribosome binding site sequence "AGGAGGUAAAACAU" was prepended before the AUG start codon when determining the folding free energies, as was the case for the actual genes. For each variant, a Pearson's correlation coefficient "r", and the root mean square deviation "RMSD" are given as measures of similarities of their folding free energy profiles to the wild-type sequence.
