# Supplementary material for: Inferring gene function from evolutionary change in signatures of translation efficiency
Source: Genome Biol. 2014 Mar 3;15(3):R44. doi: 10.1186/gb-2014-15-3-r44 (PMC4054840; doi:10.1186/gb-2014-15-3-r44)
Supplement: Additional file 15 — Distributions of predicted functional interactions at different confidence levels. Functional interactions were examined between (1) 30 clusters of orthologous groups (COGs) known to have a role in the oxidative stress response, labeled ‘known versus known’; (2) the ‘known’ group and the 34 COGs found to be differentially expressed between aerotolerant organisms and anaerobes, or between obligate and facultative aerobes, labeled ‘diffExpr versus known’; and (3) the ‘known’ group and a 100 randomly chosen COGs, labeled ‘randomSet versus known’. Two of the 34 COGs were also in the ‘known’ group, and their functional interactions did not count for the ‘diffExpr’ group; these were COG0719 (E. coli sufD and sufB genes) and COG1249 (E. coli lpd, ykgC, gor, and sthA genes). The predicted functional interactions are from the STRING v9.0 database [39], the scores vary from 0 to 1; STRING declares interactions between 0.15 and 0.40 to have low confidence, between 0.40 and 0.70 to have medium confidence, and above 0.70 to have high confidence. For details on how the scores are computed for each individual source of data, please refer to references given at the STRING website. The P values are from a χ2 test. [file gb-2014-15-3-r44-S15.docx]

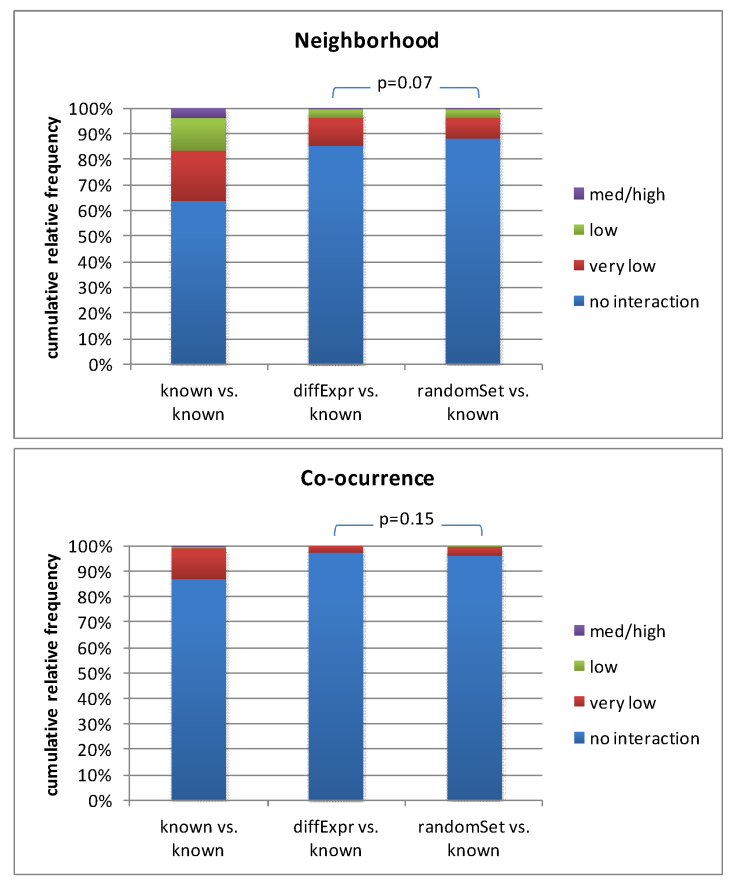


**Additional file 15. Distributions of predicted functional interactions at different confidence levels.** Functional interactions were examined between (a) 30 COGs known to have a role in the oxidative stress response, labeled "known *vs.* known"; (b) the "known" group and the 34 COGs found to be differentially expressed between aerotolerant organisms and anaerobes, or between obligate and facultative aerobes, labeled "diffExpr *vs.* known"; (c) the "known" group and a 100 randomly chosen COGs, labeled "randomSet *vs.* known". Two of the 34 COGs were also in the "known" group, and their functional interactions did not count for the "diffExpr" group. In particular: COG0719 (*E. coli* *sufD* and *sufB* genes) and COG1249 (*E. coli* *lpd*, *ykgC*, *gor* and *sthA* genes). The predicted functional interactions are from the STRING v9.0 database, http://string-db.org/; the scores vary from 0 to 1, where STRING declares interactions betwen 0.15 and 0.40 to have low confidence, between 0.40 and 0.70 medium, and above 0.70 high confidence. For details on how the scores are computed for each individual source of data, please refer to the papers describing the database (references given at the STRING website). The *P* values were from a χ^2^ test.
