# Supplementary material for: Inferring gene function from evolutionary change in signatures of translation efficiency
Source: Genome Biol. 2014 Mar 3;15(3):R44. doi: 10.1186/gb-2014-15-3-r44 (PMC4054840; doi:10.1186/gb-2014-15-3-r44)
Supplement: Additional file 16 — Relationships of the aerotolerance phenotype with the presence/absence patterns and with the codon adaptation of the catalase genes. Tables show the count of organisms (not genes) with the clusters of orthologous groups (COGs) being absent (first column), present with one or more genes that are all non-highly expressed (HE) (second column), or present with one or more genes of which at least one in the genome is HE (third column). The tables below show the same frequencies, but normalized to the total number of aerotolerant or strictly anaerobic organisms. For both COGs, the presence of the catalases in the genome is strongly and significantly correlated with aerobicity (top right panel for each COG). However, the codon adaptation of the catalases is strongly but not significantly correlated with aerobicity (bottom right panel for each COG), because of the low numbers of strictly anaerobic genomes that have a catalase gene present. [file gb-2014-15-3-r44-S16.docx]

**Additional file 16. Relationships of the aerotolerance phenotype with the presence/absence patterns and with the codon adaptation of the catalase genes.** Tables show the count of organisms (not genes) which have the COG absent (first column), present with one or more genes which are all non-HE (second column), or present with one or more genes of which at least one in the genome is HE (third column). The tables below show the same frequencies, but normalized to the total number of aerotolerant or strictly anaerobic organisms. For both COGs, the presence of the catalases in the genome is strongly and significantly correlated with aerobicity (top right panel for each COG). However, the codon adaptation of the catalases is strongly but not significantly correlated with aerobicity (bottom right panel for each COG) due to low numbers of strictly anaerobic genomes that have a catalase gene present.

| **COG:0376 (*katG*, *katP* in *E. coli*)** | | | |  |  |  |
| --- | --- | --- | --- | --- | --- | --- |
| # genomes | absent | present, not HE | present, HE |  | ***absent vs. present*** | |
| aerotolerant (n=514) | 300 | 189 | 25 |  | relative risk = 3.07  (95% CI: 2.16 to 4.37) | |
| strictly anaerobic (n=214) | 185 | 28 | 1 |  | Fisher's exact test P = 2.5e-14 | |
|  |  |  |  |  |  |  |
| *(normalized per row)* | absent | present, not HE | present, HE |  | ***HE vs. non-HE (when present)*** | |
| aerotolerant (n=514) | 58.4% | 36.8% | 4.9% |  | relative risk = 3.39  (95% CI: 0.48 to 24.07) | |
| strictly anaerobic (n=214) | 86.4% | 13.1% | 0.5% |  | Fisher's exact test P = 0.33 | |
|  |  |  |  |  |  |  |
|  |  |  |  |  |  |  |
| **COG:0753 (*katE* in *E. coli*)** | | | |  |  |  |
| # genomes | absent | present, not HE | present, HE |  | ***absent vs. present*** | |
| aerotolerant (*n*=514) | 219 | 254 | 41 |  | relative risk = 3.41  (95% CI: 2.51 to 4.64) | |
| strictly anaerobic (*n*=214) | 178 | 35 | 1 |  | Fisher's exact test P = 4.2e-25 | |
|  |  |  |  |  |  |  |
| *(normalized per row)* | absent | present, not HE | present, HE |  | ***HE vs. non-HE (when present)*** | |
| aerotolerant (*n*=514) | 42.6% | 49.4% | 8.0% |  | relative risk = 5.00  (95% CI: 0.71 to 35.28) | |
| strictly anaerobic (*n*=214) | 83.2% | 16.4% | 0.5% |  | Fisher's exact test P = 0.064 | |
