# Supplementary material for: Inferring gene function from evolutionary change in signatures of translation efficiency
Source: Genome Biol. 2014 Mar 3;15(3):R44. doi: 10.1186/gb-2014-15-3-r44 (PMC4054840; doi:10.1186/gb-2014-15-3-r44)
Supplement: Additional file 17 — A cross-validation test of the ability to retrieve functionally related genes, starting from the translational efficiency profiles of clusters of orthologous groups (COGs) across genomes (left panel), or the gene presence/absence profiles (right panel, equivalent to a standard phyletic profiling approach). The test uses Escherichia coli K12 genes that are assigned to a COG and that are annotated with one of the five Gene Ontology (GO) categories above, and compares these genes with a sample of other E. coli genes that are in COGs but that do not have this GO function assigned. The size of the sample of these ‘negative genes’ is 19 times the number of ‘positive’ genes, which thus make up 5% of the combined dataset, mimicking a realistic distribution. Next, a Random Forest model is trained to discriminate the two groups of E. coli genes, and tested in a n-fold cross-validation scheme (using Weka 3.7.9), where n is the number of positive genes for that GO. The plots are precision-recall curves: recall is on the x axis, precision on the y axis. Importantly, the translation efficiency models (left panel) do not have access to gene presence/absence information, and must discriminate the groups only from the codon biases of the present genes; absent genes are encoded as missing data. The measure of translation efficiency in the profiles is the difference in classifier probabilities of the intergenic DNA versus codon usage data (Figure 1A, left versus right). [file gb-2014-15-3-r44-S17.pdf]

translation efficiency profile

	g1	g2	g3	g4	g5	g6	g7	g8	g9	g10
COG1	0.1		0.8			0.2		0.1		0.9
COG2	0.3					0.3	0.8	0.2		1
COG3	0.2		0.9			0.2	0.9	0.3		0.9
COG4	0.3		1			0.1	0.9		1	1
COG5	0.1		0.9			0.2	0.8	0.2		0.8
COG6		0.8			0.4				0.2	
COG7	0.9			0.3	0.5		0	0.9	0.1	0
COG8		0.9	0.1		0.4	0.9			0.2	
COG9	1	0.7			0.6	1	0.1		0.1	0
COG10		0.9	0.1	0.4	0.5		0	0.9	0.2	

(table for illustration purposes only)

standard phyletic profile

	g1	g2	g3	g4	g5	g6	g7	g8	g9	g10
COG1	1	0	1	0	0	1	0	1	0	1
COG2	1	0	0	0	0	1	1	1	0	1
COG3	1	0	1	0	0	1	1	1	0	1
COG4	1	0	1	0	0	1	1	0	1	1
COG5	1	0	1	0	0	1	1	1	0	1
COG6	0	1	0	0	1	0	0	0	1	0
COG7	1	0	0	1	1	0	1	1	1	1
COG8	0	1	1	0	1	1	0	0	1	0
COG9	1	1	0	0	1	1	1	0	1	1
COG10	0	1	1	1	1	0	1	1	1	0

(table for illustration purposes only)

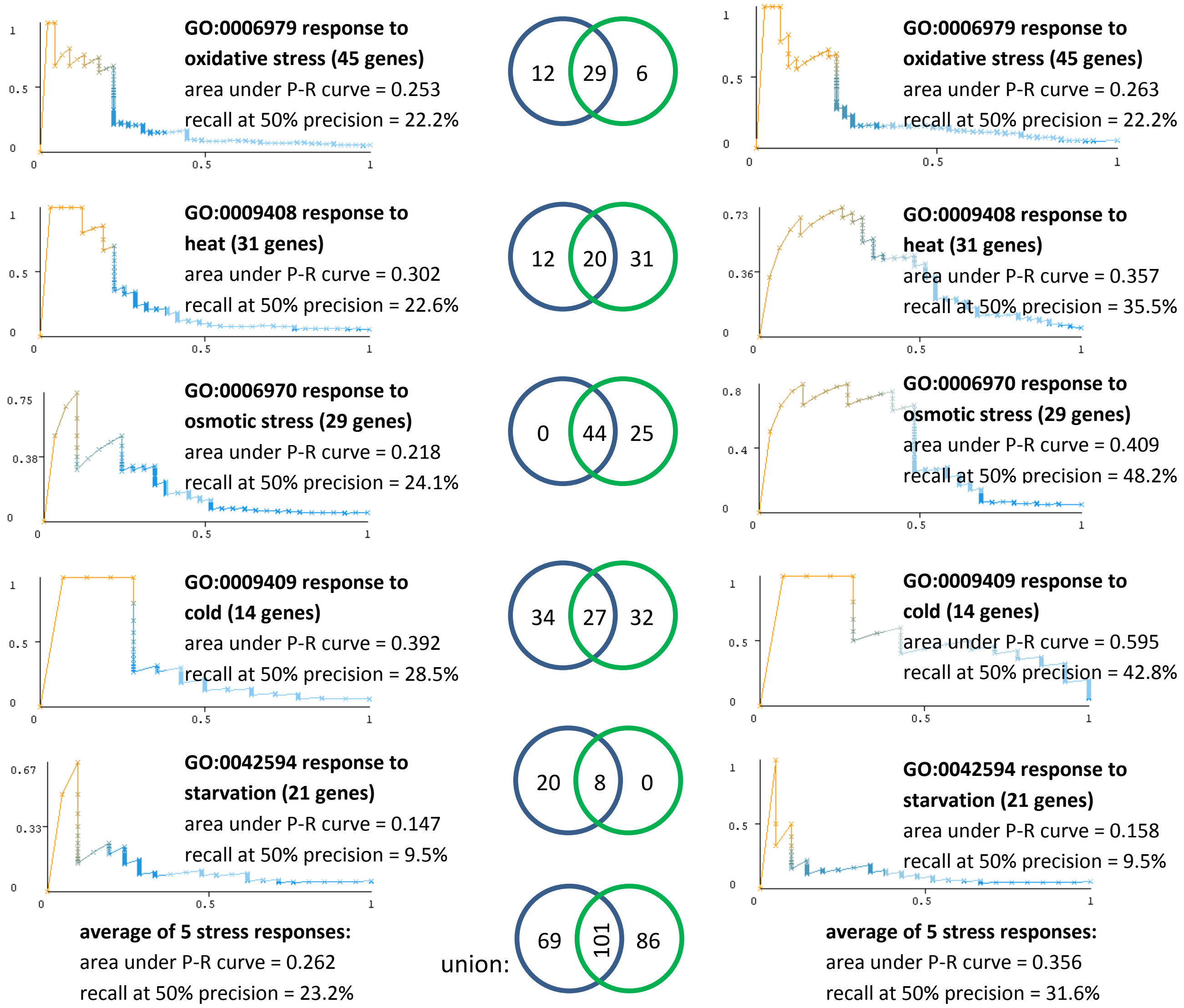

Additional file 17. A cross-validation test of the ability to retrieve functionally related genes, starting from the translational efficiency profiles of COGs across genomes (left panel), or the gene presence/absence profiles (right panel, equivalent to a standard phyletic profiling approach). The test uses *E. coli* K12 genes that are assigned to a COG and that are annotated with one of the five GO categories above, and compares these genes to a sample of other *E. coli* genes in COGs, but that do not have this GO function assigned. The size of the sample of these 'negative genes' is 19x the number of 'positive' genes, which thus make up 5% of the combined dataset, mimicking a realistic distribution. Then, a Random Forest model is trained to discriminate the two groups of *E. coli* genes, and tested in a *n*-fold crossvalidation scheme (RF in Weka 3.7.9, I=1000, K=30), where *n* is the number of positive genes for that GO. The plots are precision-recall curves: recall is on *x* axis, precision on *y*. Importantly, the "translation efficiency" models (left panel) do not have access to gene presence/absence information and must discriminate the groups only from the codon adaptation of the present genes; absent genes are encoded as missing data. The measure of translation efficiency in the profiles is the difference of classifier probabilities of the intergenic DNA vs. codon usage data (Fig 1A, left *vs*. right). Venn diagrams show the # genes with a newly predicted function when applying the crossvalidated models to the complete *E. coli* genome (3534 genes in COGs with a sufficient phylogenetic representation); left circle = translation efficiency profile, right = phyletic profile; both models were applied at a confidence threshold corresponding to 50% precision.
