# Supplementary material for: Inferring gene function from evolutionary change in signatures of translation efficiency
Source: Genome Biol. 2014 Mar 3;15(3):R44. doi: 10.1186/gb-2014-15-3-r44 (PMC4054840; doi:10.1186/gb-2014-15-3-r44)
Supplement: Additional file 20 — Designed variants of Escherichia coli clpS and yjjB genes, with progressively more optimal codons replaced by suboptimal ones (Figure 6). The lowercase ‘a’ in the yjjB sequences denotes a replacement of the original G with an A to abolish a HsdR site. [file gb-2014-15-3-r44-S20.docx]

**Additional file 20. Designed variants of *E. coli* *clpS* and *yjjB* genes, with progressively more optimal codons replaced by suboptimal ones (Fig. 6).** The lowercase "a" in the *yjjB* sequences denotes a replacement of the original G with an A to abolish a HsdR site.

| **ID** | **DNA** |
| --- | --- |
| *clpS*_w.t. | ATGGGTAAAACGAACGACTGGCTGGACTTTGATCAACTGGCGGAAGAAAAAGTTCGCGACGCGCTAAAACCGCCATCTATGTATAAAGTGATATTAGTCAATGATGATTACACTCCGATGGAGTTTGTTATTGACGTGTTACAAAAATTCTTTTCTTATGATGTAGAACGTGCAACGCAATTGATGCTCGCTGTTCACTACCAGGGGAAGGCCATTTGCGGAGTCTTTACCGCCGAGGTTGCAGAAACCAAAGTGGCGATGGTGAACAAGTACGCGAGGGAGAATGAGCATCCATTGCTGTGTACGCTAGAAAAAGCCTGA |
| *clpS*_15 | ATGGGTAAGACGAACGACTGGCTGGACTTTGATCAACTTGCGGAGGAGAAAGTTCGCGACGCGCTAAAGCCGCCATCTATGTATAAAGTGATATTAGTCAATGATGATTACACTCCTATGGAGTTTGTTATTGACGTGTTACAAAAATTTTTTAGTTATGATGTAGAACGCGCAACGCAATTGATGCTCGCTGTTCATTATCAGGGGAAGGCCATTTGCGGAGTCTTTACGGCCGAGGTTGCAGAGACCAAAGTGGCGATGGTGAATAAGTACGCGAGGGAGAATGAGCATCCATTGCTGTGTACGCTAGAGAAAGCCTGA |
| *clpS*_20 | ATGGGTAAGACGAACGACTGGCTGGACTTTGATCAACTTGCGGAGGAGAAAGTTCGCGACGCGCTAAAGCCACCATCTATGTATAAAGTGATATTAGTCAATGATGATTATACTCCTATGGAGTTTGTTATTGACGTGTTACAAAAATTTTTTAGTTATGATGTAGAACGCGCAACGCAATTGATGCTCGCTGTGCATTATCAGGGGAAGGCCATTTGTGGAGTCTTTACGGCCGAGGTTGCAGAGACCAAAGTGGCGATGGTGAATAAGTACGCGAGGGAGAATGAGCATCCATTGCTGTGTACGCTAGAGAAGGCCTGA |
| *clpS*_25 | ATGGGTAAGACGAATGACTGGTTAGACTTTGATCAACTTGCGGAGGAGAAAGTTCGCGACGCGCTAAAGCCACCATCTATGTATAAAGTGATATTAGTCAATGATGATTATACTCCTATGGAGTTTGTTATTGACGTGTTACAAAAGTTTTTTAGTTATGATGTAGAACGCGCAACGCAATTGATGCTCGCTGTGCATTATCAAGGGAAGGCCATTTGTGGAGTCTTTACGGCCGAGGTTGCAGAGACCAAAGTGGCGATGGTGAATAAGTACGCGAGGGAGAATGAGCATCCATTGCTCTGTACGCTAGAGAAGGCCTGA |
| *yjjB_*w.t. | ATGGGTGTGATCGAATTTCTGTTAGCGTTGGCGCAGGATATGATCCTCGCCGCCATTCCTGCGGTCGGCTTTGCGATGGTGTTCAACGTTCCCGTaCGGGCGTTACGCTGGTGTGCGCTGCTTGGCTCGATAGGTCATGGTTCCCGAATGATCTTGATGACCAGCGGGTTGAATATTGAGTGGTCAACCTTTATGGCTTCTATGCTGGTCGGTACCATTGGTATTCAATGGTCGCGCTGGTATCTGGCGCATCCGAAAGTGTTTACCGTGGCGGCCGTTATCCCTATGTTCCCGGGCATATCGGCTTATACCGCAATGATTTCGGCGGTAAAAATCAGCCAGTTAGGTTACAGCGAACCGTTGATGATTACCCTGTTAACCAACTTTCTTACAGCTTCATCGATTGTTGGTGCGTTATCCATCGGTCTTTCCATTCCTGGATTATGGTTGTACCGCAAGCGCCCTCGCGTATAA |
| *yjjB*_21 | ATGGGTGTGATCGAGTTTCTGTTAGCGTTGGCGCAGGATATGATCCTCGCCGCCATTCCTGCGGTCGGCTTTGCGATGGTGTTTAATGTTCCCGTaCGGGCGTTACGCTGGTGTGCGCTCCTTGGCTCGATAGGTCATGGTTCCCGAATGATCTTGATGACCAGCGGGTTGAATATTGAGTGGTCAACCTTTATGGCGAGTATGCTGGTCGGTACCATTGGTATTCAATGGTCGCGCTGGTATTTAGCGCATCCTAAGGTGTTTACAGTGGCGGCCGTCATCCCTATGTTTCCGGGCATATCGGCTTATACCGCAATGATTTCGGCGGTAAAAATTAGCCAATTAGGTTATAGCGAGCCATTGATGATTACGTTATTAACGAACTTTCTTACAGCTTCATCGATTGTCGGTGCGTTATCCATCGGTCTTTCCATTCCTGGATTATGGTTGTACCGCAAGCGCCCTCGCGTATAA |
| *yjjB*_28 | ATGGGTGTGATCGAGTTTCTGTTAGCGTTGGCGCAGGATATGATCCTCGCCGCCATTCCTGCGGTCGGCTTTGCGATGGTGTTTAATGTCCCCGTaCGGGCGTTACGCTGGTGTGCGCTCCTTGGCTCGATAGGTCATGGTTCCCGAATGATCTTGATGACCAGCGGGTTGAATATTGAGTGGTCAACCTTTATGGCGAGTATGCTTGTCGGTACCATTGGTATTCAATGGTCGCGCTGGTATTTAGCGCATCCTAAGGTGTTTACAGTGGCGGCCGTCATTCCTATGTTTCCGGGCATATCGGCTTATACCGCAATGATTTCGGCGGTAAAGATTAGCCAATTAGGTTATAGCGAGCCATTGATGATTACGTTATTAACGAACTTTCTTACAGCTTCATCGATTGTCGGTGCGTTATCCATTGGTCTTTCCATTCCTGGATTATGGTTGTATCGCAAGCGCCCTCGCGTATGA |
| *yjjB*_35 | ATGGGTGTGATTGAGTTTTTGTTAGCGTTGGCGCAGGATATGATCCTCGCCGCCATTCCTGCGGTCGGCTTTGCGATGGTGTTTAATGTCCCCGTaCGGGCGTTACGCTGGTGTGCGCTCCTTGGCTCGATAGGTCATGGTTCCCGAATGATTTTGATGACCAGCGGGTTGAATATTGAGTGGTCAACGTTTATGGCGAGTATGCTTGTCGGTACCATTGGTATTCAATGGTCGCGCTGGTATTTAGCGCATCCTAAGGTGTTTACAGTGGCGGCCGTCATTCCTATGTTTCCAGGCATATCGGCCTATACCGCAATGATTTCGGCGGTAAAGATTAGCCAATTAGGTTATAGCGAGCCATTGATGATTACGTTATTAACGAATTTTCTTACAGCTTCATCGATTGTCGGTGCGTTATCCATTGGTCTTTCCATTCCTGGATTATGGTTGTATCGCAAGCGCCCTCGCGTATGA |
